# Generative spatial artificial intelligence for sustainable smart cities: A pioneering large flow model for urban digital twin

**DOI:** 10.1016/j.ese.2025.100526

**Published:** 2025-01-15

**Authors:** Jeffrey Huang, Simon Elias Bibri, Paul Keel

**Affiliations:** Institute of Computer and Communication Sciences (IINFCOM), School of Architecture, Civil and Environmental Engineering (ENAC), Media and Design Laboratory (LDM), Swiss Federal Institute of Technology Lausanne (EPFL), 1015, Lausanne, Switzerland

**Keywords:** Sustainable smart cities, Generative artificial intelligence, Generative spatial artificial intelligence, Foundation models, Large flow model, Urban digital twin, Urban planning and design

## Abstract

Rapid urbanization, alongside escalating resource depletion and ecological degradation, underscores the critical need for innovative urban development solutions. In response, sustainable smart cities are increasingly turning to cutting-edge technologies—such as Generative Artificial Intelligence (GenAI), Foundation Models (FMs), and Urban Digital Twin (UDT) frameworks—to transform urban planning and design practices. These transformative tools provide advanced capabilities to analyze complex urban systems, optimize resource management, and enable evidence-based decision-making. Despite recent progress, research on integrating GenAI and FMs into UDT frameworks remains scant, leaving gaps in our ability to capture complex urban flows and multimodal dynamics essential to achieving environmental sustainability goals. Moreover, the lack of a robust theoretical foundation and real-world operationalization of these tools hampers comprehensive modeling and practical adoption. This study introduces a pioneering Large Flow Model (LFM), grounded in a robust foundational framework and designed with GenAI capabilities. It is specifically tailored for integration into UDT systems to enhance predictive analytics, adaptive learning, and complex data management functionalities. To validate its applicability and relevance, the Blue City Project in Lausanne City is examined as a case study, showcasing the ability of the LFM to effectively model and analyze urban flows—namely mobility, goods, energy, waste, materials, and biodiversity—critical to advancing environmental sustainability. This study highlights how the LFM addresses the spatial challenges inherent in current UDT frameworks. The LFM demonstrates its novelty in comprehensive urban modeling and analysis by completing impartial city data, estimating flow data in new locations, predicting the evolution of flow data, and offering a holistic understanding of urban dynamics and their interconnections. The model enhances decision-making processes, supports evidence-based planning and design, fosters integrated development strategies, and enables the development of more efficient, resilient, and sustainable urban environments. This research advances both the theoretical and practical dimensions of AI-driven, environmentally sustainable urban development by operationalizing GenAI and FMs within UDT frameworks. It provides sophisticated tools and valuable insights for urban planners, designers, policymakers, and researchers to address the complexities of modern cities and accelerate the transition towards sustainable urban futures.

## Highlights

• The study introduces the Large Flow Model (LFM) specifically designed for integration into Urban Digital Twin (UDT) systems.
• It integrates Generative AI and Foundation Models to advance sustainable smart city planning and design through UDT.
• The LFM demonstrates the ability to analyze and model complex urban flows and multimodal dynamics.
• It showcases its novelty by completing city data, estimating urban flows, predicting their evolution, and revealing urban dynamics.
• The research contributes AI-driven solutions for creating efficient, resilient, and sustainable urban environments.

## Graphical abstract

**Figure:**
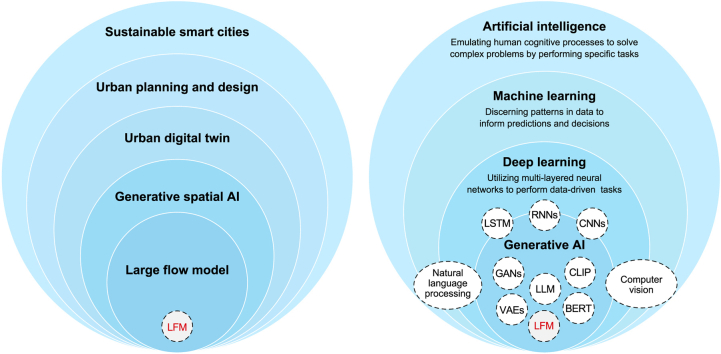

## 1 Introduction

Sustainable urban development has long been a key focus in addressing the growing challenges posed by rapid urbanization, resource depletion, and ecological degradation. These intertwined problems demand holistic solutions that mitigate their impacts and leverage opportunities for creating smarter and more sustainable urban environments. To tackle these complex challenges and problems, sustainable smart cities are increasingly leveraging cutting-edge technologies and embracing innovative strategies for achieving environmental sustainability goals. At the forefront of these advancements, Artificial Intelligence (AI) and Urban Digital Twin (UDT) technologies stand out for their transformative capabilities (e.g., Refs. [[1], [2], [3], [4], [5], [6], [7], [8]]), particularly in driving innovation in and for environmental planning and design within the dynamic landscape of sustainable smart cities [[9], [10], [11]]. An UDT is a dynamic, virtual representation of urban environments that integrates real-time data and advanced modeling techniques to simulate, analyze, predict, and optimize urban systems. In this study, UDT serves as a platform for modeling complex urban flows, facilitating data-driven decision-making for sustainable smart city planning and design. Complementing UDT, AI encompasses a broad spectrum of sophisticated models and algorithms that enable computer systems to emulate human cognitive processes, such as learning, reasoning, and problem-solving. For this research, AI enhances UDT's analytical and predictive capabilities of UDT by analyzing complex urban data, modeling interconnected systems, and generating actionable insights for enhancing environmental planning and design practices in sustainable smart cities.

A diverse range of models and techniques from various AI subdomains are being increasingly applied to transform urban planning (e.g., Ref. [[12], [13], [14], [15], [16]]) and design [[17], [18], [19]] in sustainable smart cities [9]. In the realm of UDT, AI drives the creation of dynamic, real-time city models that support predictive analytics, scenario testing, and system optimization [[20], [21], [22], [23]]. Predictive analytics is critical for proactive planning and design, enabling cities to adapt to evolving challenges, identify bottlenecks, and enhance urban efficiency. Moreover, AI-driven UDT enables the anticipation of urban trends, the evaluation of planning strategies, and the identification of optimal solutions, providing a comprehensive foundation for adaptive and sustainable urban systems. Its capabilities enhance urban dynamics [24] and strengthen urban resilience [25,26], empowering planners, designers, and stakeholders to make evidence-based decisions to promote the vision of sustainable smart cities. Furthermore, by addressing critical aspects of environmental sustainability—such as resource allocation, infrastructure development, transportation optimization, pollution reduction, energy management, and biodiversity conservation—AI-driven UDT provides a comprehensive framework for optimizing and planning these urban environments (see Ref. [11] for a detailed review).

In light of the above, recent advancements in AI subfields—particularly Machine Learning (ML), Deep Learning (DL), Computer Vision (CV), and Natural Language Processing (NLP)—are increasingly being adopted by sustainable smart cities to tackle the wicked problems associated with planning and design within the UDT framework. Integrating AI models from these subfields empowers planners and designers to address intricate tasks by utilizing AI-driven analytical tools or UDT systems. These applications include managing and analyzing vast datasets, uncovering patterns, and generating actionable insights to enhance decision-making processes across multiple urban domains.

In addition to the common AI subfields or subdomains, the advent of Generative AI (GenAI) represents a new frontier to transform urban planning [[27], [28], [29], [30], [31], [32]] and design [[32], [33], [34], [35], [36], [37], [38], [39], [40]] processes and practices. GenAI is a class of AI models that create new content, such as images, text, code, or simulations, by learning patterns from existing data. Within this work, GenAI is leveraged to enhance UDT functionalities by generating realistic urban flow data and scenarios and providing predictive insights. GenAI models, such as Generative Adversarial Networks (GANs) (e.g., Refs. [41,42]), Variational Autoencoders (VAEs), (e.g., Ref. [[43], [44], [45]]), Transformers (e.g., Ref. [[46], [47], [48]]), and Diffusion Models (DM) (e.g., Ref. [49,50]), enable the automatic generation of design alternatives and scenarios, fostering creativity and innovation in city planning and design while enhancing the capabilities of UDT to simulate diverse urban systems and explore various urban futures. They are progressively integrated into UDT's modeling and simulation functionalities (e.g., Ref. [[51], [52], [53]]). Although their practical application in UDT within the context of urban planning and design remains limited, it is evolving rapidly [9,29,54], signaling a promising future where these tools could play a crucial role in transforming urban environments into more efficient, sustainable, and livable spaces.

Moreover, AI is undergoing a significant shift with the emergence and adoption of Foundation Models (FMs) or Pre-trained Foundation Models (PFMs). The difference between FMs and PFMs lies in their training status and how they are positioned for further application or use. FMs are large-scale AI models trained on massive amounts of diverse data. They are designed to be task-agnostic, meaning they are not specialized for a particular application during initial training but can be adapted for many downstream tasks (see, e.g., Ref. [55]; Mai, 2022, 2024; Xie et a., 2023; [56]). PFMs refer specifically to FMs that have already undergone extensive pre-training on large, diverse datasets. They are ready for downstream applications or fine-tuning for specific tasks, making them practical tools for solving real-world problems without needing massive computational resources to train from scratch (see, e.g., Ref. [46,[57], [58], [59]]). Further, as large-scale neural networks, FMs use advanced architectures to learn general-purpose features and representations, which can be adapted to perform a wide range of specific tasks (e.g., Ref. [32,37,[60], [61], [62]]), such as text generation, code generation, image generation, and knowledge graph construction. These examples highlight the versatility of FMs, showcasing their potential to drive advancements in GenAI applications across diverse domains. As a broader category of models, FMs can include GenAI as a subset, a specialized application of AI that often leverages the capabilities of FMs for generating novel data. While GenAI models can technically perform without FMs, their performance and versatility may be limited, especially in tasks requiring a large amount of data or prior knowledge.

Furthermore, the potential integration of GenAI and FMs with UDT—given their synergistic computational and analytical capabilities—presents promising opportunities to revolutionize city planning and design. Specifically, incorporating GenAI and FMs into the functionalities of UDT is viewed as a strategic approach to addressing the critical challenges faced by its existing computational processes and simulation models. These challenges include limited scenario exploration, analysis and interpretation of unstructured data, decision-making support (alternative solutions), complexity and integration, realism and visualization [8,[63], [64], [65], [66], [67]], inadequate data availability [68,69], data inconsistency and inaccuracy (e.g., Ref. [65,68,70,71]), and computational constraints requiring multilevel integrated models [72,73].

The focal point of this study is the need for a more enhanced and nuanced approach to managing spatial data, conducting analysis, and performing simulations within the UDT framework. This stems from the inherent limitations of current UDT frameworks, particularly their challenges in effectively capturing spatial dynamics and space vitality, both of which are essential for urban planning and design [21,[74], [75], [76], [77]]. Additionally, the complexities of urban flows, which include the movement of people, goods, energy, and information, require advanced models to accurately simulate and predict these interrelated dynamics to inform effective urban management and decision-making. Without addressing these challenges, UDT may struggle to provide accurate representations of urban systems and precise predictions, hindering the ability of planners and designers to make informed decisions and enhance sustainable development practices. Therefore, incorporating GenAI and FMs into UDT aims to enhance its computational and analytical capabilities, allowing for more comprehensive and insightful data-driven analyses and simulations to propel sustainable and resilient urban development.

GenAI can reduce the costs, time, and labor associated with collecting scenario-specific data, generating relevant scenarios, creating three-dimensional (3D) city models, and improving multi-scale urban design processes for predictive analytics and decision-making [54]. Moreover, FMs offer powerful tools for advancing sustainable smart cities by transforming how they are envisioned, designed, and planned, enabling more efficient and data-driven urban development strategies and practices. FMs play a crucial role in advancing GenAI and present vast opportunities and challenges across multiple domains (e.g., Ref. [[78], [79], [80]]). In particular, the pre-training phase in FMs allows them to capture rich representations of the input data, which can then be fine-tuned on labeled datasets for specific domains [60,62,79]. By fine-tuning FMs on these tasks or domains, state-of-the-art performance can be achieved with minimal data and computational resources [55,60,79,81]. FMs represent unprecedented opportunities to enhance urban planning and design practices, leading to smarter, more resilient and livable cities.

However, despite recent progress in GenAI and FMs, significant gaps remain in their integration into UDT frameworks as data-driven urban planning and design systems. Specifically, there is a limited understanding of how these emerging technologies can enhance decision-making processes by effectively capturing critical urban flows—such as mobility, goods, energy, waste, materials, and biodiversity—to advance environmental goals in sustainable smart cities. Integration challenges, including harmonizing diverse urban data systems, developing scalable architectures, and ensuring system interoperability, underscore the complexity of implementing FMs, GenAI, and UDT in real-world urban settings. Research on operationalizing these emerging technologies as a unified system remains scant. This gap emphasizes the urgent need for practical methodologies tailored to urban flows and spatial dynamics, essential for accelerating the development and implementation of effective UDT solutions.

From a theoretical perspective, existing scholarly frameworks in sustainable smart city planning and design and UDT have recently laid the groundwork for integrating GenAI and FMs into data-driven decision-making processes. However, these efforts remain predominantly conceptual and exploratory in nature, as they are still in the early stages of development, with researchers working to refine and expand their scope [9,54,82]. A notable gap in current frameworks is the absence of a structured theoretical foundation to guide the integration of GenAI and FMs into UDT systems, limiting the ability to address the complexities of urban flows and multimodal dynamics, especially in modeling and analyzing critical domains essential for advancing environmental sustainability goals.

To address the identified gaps, this study introduces a pioneering Large Flow Model (LFM), grounded in a robust foundational framework and designed with GenAI capabilities. It is specifically tailored for integration into UDT systems to enhance predictive analytics, adaptive learning, and complex data management functionalities. This advancement aims to drive progress in sustainable smart city planning and design. To validate its applicability and relevance, the Blue City Project in Lausanne City is examined as a case study, showcasing the ability of the LFM to effectively model and analyze urban flows and address the spatial challenges associated with current UDT frameworks. Given the interconnected nature and developmental stage of the subprojects within the Blue City initiative, this study focuses primarily on the design and ongoing development phases of the LFM, with implementation and testing planned as subsequent steps in the progression towards the operational LFM.

The development of the LFM is anchored by two interrelated elements: the foundational framework and the conceptual design. The former serves as the overarching theoretical basis, organizing the core ideas, structural relationships, and objectives that guide the model's development. Operating at a high level, it addresses the "why" and "what" of the LFM's existence, focusing on the guiding principles and relationships necessary for achieving its intended goals. In contrast, the latter translates this theoretical foundation into a practical, functional model. It focuses on the "how" of implementing the ideas outlined in the foundational framework, detailing the structuring of the LFM's components and their functionality. It specifies actionable elements such as workflows, processes, and tools for real-world application by operationalizing theoretical relationships. Together, the foundational framework and the conceptual design create a cohesive strategy that ensures the LFM is theoretically sound and practically applicable.

The remainder of this study is structured as follows: Section 2 presents a state-of-the-art review that analyzes and synthesizes the conceptual and practical foundations of the study, culminating in a foundational framework developed from the insights gained through this review. Section 3 provides a comprehensive overview of the Blue City Project, along with its eight interconnected subprojects. It employs this initiative as a case study to elucidate the project's scope, progress, and impact, while explaining this study’s association with Subproject 8. Section 4 outlines the research methodology, focusing on the design and ongoing development phases of the LFM. Section 5 introduces the pioneering LFM, detailing these two phases as key results. Section 6 provides a comprehensive discussion, encompassing a summary of findings and their interpretation, a comparative analysis, implications, limitations, challenges, recommendations for future research and development, and an account of the positioning, scope, and scalability of the LFM. Finally, Section 7 concludes the study by summarizing the findings, highlighting key contributions, and offering closing remarks.

## 2 State-of-the-art review: conceptual and practical foundations

This section lays the groundwork for the study by exploring the intersection of AI and its subfields with urban planning and design and UDT frameworks in the dynamic context of sustainable smart cities. It highlights the transformative roles of ML, DL, CV, NLP, and GenAI, focusing on the potential of GenAI and FMs. The review provides theoretical and practical foundations, setting the stage for developing the foundational framework for the LFM. By examining how GenAI and FMs can enhance decision-making and drive innovation in urban planning and design through their integration with UDT frameworks, the section identifies key research gaps and opportunities for advancing sustainable urban development.

### 2.1 Thematic literature review and its structured process

A thematic literature review is a structured approach to examining existing research, focusing on identifying, analyzing, and synthesizing recurring themes or patterns within the literature. This approach organizes the body of work into central themes or categories, providing a cohesive narrative that highlights trends, synthesizes findings, and identifies gaps in knowledge. Often used as a foundational step in research or framework development, a thematic literature review categorizes prior studies under thematic headings and integrates insights from these categories to inform conceptual framework development. This methodological approach is particularly valuable for addressing complex, interdisciplinary topics, as it provides a structured pathway for connecting diverse findings into a unified understanding.

This subsection outlines the primary approach to developing a conceptual framework for the LFM tailored for UDT. This review focuses on synthesizing insights and findings from recent theoretical and empirical studies on AI, GenAI, FMs, and UDT applications within the dynamic context of sustainable smart city planning and design. It serves as the basis for identifying research gaps and informing the conceptual design of the LFM within the Generative Spatial AI (GSAI) framework. By addressing these gaps, the study positions the LFM as a pioneering approach for modeling complex urban flows and enhancing decision-making processes in urban planning and design. Here, the conceptual design refers to how the foundational framework guides the definition of the specific architecture and functionality of the LFM, as detailed in Section 6. This distinction ensures a clear connection between the theoretical underpinnings developed through the thematic literature review and their practical application in the proposed model.

The thematic literature review explores the integration of AI, GenAI, and FMs with UDT, highlighting their transformative potential in advancing sustainable urban development through planning and design. This review provides the foundation for the conceptual framework and ensures alignment with the state-of-the-art in the field by identifying potential applications and critical challenges. It expands on the discussion in the introduction, drawing upon its foundational insights to contextualize the study and underscore its relevance within the broader scientific discourse.

**Fig. 1:**
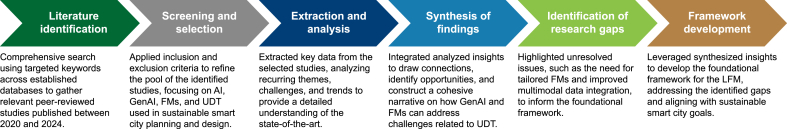
A flow diagram outlining the step-by-step process of developing the foundational framework.

The review followed a structured process (Fig. 1) to ensure the inclusion of relevant and high-quality studies. Peer-reviewed journal articles, conference proceedings, and books published primarily between 2020 and 2024 were sourced from established academic databases, including Scopus, Web of Science, ScienceDirect, and Springer Link. The search was conducted using carefully chosen keywords and their combinations to comprehensively cover the intersections among these domains. The selected keywords included combinations such as “Urban Digital Twin” OR “Digital Twin” AND “Generative Artificial Intelligence” OR “Artificial Intelligence”; “Foundation Models” OR “Pre-trained Foundation Models” AND “Urban Digital Twin” OR “Digital Twin”; “Sustainable Smart Cities” OR “Smart Cities” AND “Generative Artificial Intelligence” OR “Artificial Intelligence”; “Urban Planning” OR “Urban Design” AND “Generative Artificial Intelligence” OR “Artificial Intelligence”; and “Generative Artificial Intelligence” OR “Artificial Intelligence” AND “Environmental Sustainability” OR “Urban Sustainability.” These strategic keyword pairings ensured a thorough and targeted search, capturing the essential literature at the confluence of advanced technologies and sustainable urban development.

The inclusion criteria ensured that the selected studies offered comprehensive insights into integrating AI, GenAI, and UDTs in sustainable smart city planning and design, as well as emerging FMs. Specifically, the review prioritized studies or publications addressing the foundational principles and applications of AI and GenAI models in urban planning and design, focused on the integration of AI and GenAI with UDT as decision-support tools for sustainable smart cities, covering FMs as applied to different domains with high potential to advance sustainable urban development, highlighting environmentally sustainable and resilient smart city practices related to UDT and urban planning and design, and emphasizing the challenges and opportunities of multimodal data integration in UDT systems. Peer-reviewed articles published in English between 2020 and 2024 are primarily selected to ensure academic rigor and relevance. The review excluded studies unrelated to the focal topics, non-peer-reviewed, duplicate and redundant, and those with an overly narrow foundational and practical focus or those published before 2020.

The review ensures the inclusion of the most up-to-date developments and technological advancements in this interdisciplinary field by limiting the search to recent publications. A total of 313 articles were initially identified through the search process. After a rigorous abstract screening and the application of predefined inclusion and exclusion criteria, 127 articles were selected for final analysis. The extracted data was analyzed qualitatively to identify recurring themes and common challenges across the studies. Emerging trends in technology integration and methodological innovations were highlighted. Insights from the analyzed data were synthesized to draw connections, identify opportunities, and construct a coherent narrative, bridging theoretical underpinnings with practical applications. This synthesis process also involved integrating diverse findings to illuminate underexplored areas and provide a comprehensive understanding of the state-of-the-art at the intersection of AI, GenAI, and UDT applications in sustainable smart city planning and design and relevant insights into FMs. This approach provided a robust foundation for identifying the critical gaps and opportunities necessary for developing and enhancing the conceptual framework of the LFM, ensuring alignment with the environmental objectives of sustainable smart cities.

### 2.2 Artificial intelligence for sustainable smart city planning and design

AI refers to a branch of computer science and engineering dedicated to developing systems and algorithms capable of performing tasks that typically require human intelligence. These tasks include, but are not limited to, learning (e.g., data analysis and pattern recognition for predictive modeling in urban planning), reasoning (e.g., logical deduction and decision-making in real-time traffic management), problem-solving (e.g., optimizing energy distribution in smart grids), perception (e.g., image recognition in autonomous vehicles), and natural language understanding and generation (e.g., analyzing public feedback and extracting community sentiments for aligning urban projects with residents' needs and preferences and generating textual descriptions for city layout proposals). AI systems achieve these capabilities by leveraging various models and techniques, enabling adaptability and automation in diverse domains, including urban planning and design. Prominent subfields of AI, such as ML, DL, CV, NLP, and GenAI, provide specialized models and techniques that have been widely applied to urban planning (e.g., Ref. [[11], [12], [13], [14], [15], [16],[27], [28], [29],^31^,^32^,^83^]) and design processes [[18], [19], [32], [34], [36], [37], [38], [39], [40], [54], [84], [85]]. These advanced AI tools catalyze innovation and improve efficiency in sustainable smart city planning and design across various domains of environmental sustainability [9,10].

With their unique capabilities, ML, DL, CV, NLP, and GenAI converge to address diverse challenges in sustainable smart cities. ML provides the foundational learning framework, DL extracts intricate features from data, CV interprets visual information, NLP enables understanding and generation of human language, and GenAI enriches the process with its capability to generate novel content and adapt to evolving urban challenges, collectively enhancing the planning and design of sustainable smart cities. As evidenced by the aforementioned studies, each of these five subfields or subdomains possesses distinct AI principles, algorithms, and applications. Moreover, these subfields frequently intersect, with methodologies and techniques from one domain being adapted to solve problems in another. Indeed, they have been combined to solve more complex problems in urban planning and design (e.g., Ref. [15,18,32,37,39,83,[86], [87], [88]]; Son et al., 2022; [29,54]). For example, DL architectures, such as Generative Adversarial Networks (GANs), Convolutional Neural Networks (CNNs), Recurrent Neural Networks (RNNs), Long Short-Term Memory Networks (LSTMs), and Transformers, have become foundational for various tasks such as text summarization, language understanding, image generation, and sequence modeling [48,[89], [90], [91], [92]]. This synergy fosters collaboration and innovation, driving the development of advanced AI systems equipped to tackle complex challenges in sustainable smart city planning and design. An in-depth analysis and discussion on these aspects is provided in Bibri [9], a study that investigates the transformative potential of AI and its five subfields in advancing sustainable smart city planning and design. One notable gap identified in this study is the lack of comprehensive research on the combined applied domains of ML, DL, CV, NLP, and GenAI.

Sustainable smart cities are urban areas that prioritize environmentally friendly practices, economic prosperity, and social equity while leveraging data-driven technologies to enhance efficiency, connectivity, and the quality of life for residents. These cities aim to achieve sustainability across various dimensions, including energy use, transportation, waste management, water conservation, green spaces, digital infrastructure, and public services by integrating smart technologies, innovative policies, and community engagement. Key focus areas include [[93], [94], [95], [96], [97], [98], [99]]:

(1) Environmental sustainability: Emphasizing renewable energy sources, energy efficiency, transportation decarbonization, circular economy practices, air quality improvement, climate resilience strategies, and the protection and restoration of biodiversity and ecosystems.
(2) Economic sustainability: Promoting green job creation, fostering innovation and entrepreneurship, ensuring long-term economic growth, supporting local businesses, and reducing economic inequality through equitable resource distribution.
(3) Social Sustainability: Prioritizing affordable and accessible housing, enhancing public health and well-being, promoting cultural diversity and inclusivity, strengthening community networks, and ensuring digital inclusion by bridging the digital divide.

Addressing these interconnected dimensions enables sustainable smart cities to create resilient, inclusive, and livable urban environments that meet the needs of current and future generations while minimizing environmental impact and maximizing resource efficiency. However, the focus has recently shifted towards addressing environmental sustainability more explicitly, reflecting growing concerns about climate change, resource depletion, and biodiversity loss (e.g., Ref. [93,[100], [101], [102], [103]]). This shift underscores the urgent need for advanced AI-driven technologies and solutions that prioritize environmental sustainability goals over the socioeconomic benefits of urbanization in sustainable smart cities (e.g., Refs. [[1], [2], [3], [4], [5], [6], [7],^10^,[104], [105], [106]]).

AI models and techniques enable policymakers, planners, and designers in this rapidly evolving urban landscape to analyze vast datasets, derive valuable insights, and make informed decisions. ML models and techniques remain the most extensively utilized tools in smart cities to advance environmental sustainability goals, as evidenced by numerous studies (e.g., Ref. [1,2,[107], [108], [109], [110], [111], [112], [113], [114], [115]]) compared to DL, CV, NLP, and GenAI [1,2,104]. As these models become increasingly sophisticated and versatile, they hold immense promise for driving innovation in various domains of sustainable smart cities. Overall, the intelligence of AI lies in its capacity to iteratively learn from data, analysis results, and feedback, enhancing decision-making and optimizing outputs. This process mirrors the fundamental logic of urban planning and design practices: gathering information, analyzing it, and producing plans or designs to address environmental challenges while improving overall urban systems. Leveraging this iterative learning capability enables AI-driven approaches to adress complex problems, adapt to evolving conditions, and contribute to more informed and sustainable planning and design solutions.

### 2.3 Generative artificial intelligence models

GenAI, as a class of deep learning models, is designed to generate new data or content that mimics the characteristics of a given input data distribution by identifying and learning the underlying patterns within the data. Leveraging advanced architectures, notably GANs, VAEs, Diffusion Models, and Transformer-based models, GenAI produces realistic, coherent, and contextually appropriate outputs, including text, images, audio, code, and videos. DL, which lies at the core of GenAI, has driven significant advancements in generative models, including GANs [41,42], large-scale GANs [116], VAEs [44], Transformers [117], and flow-based models [118,119], enabling GenAI's transformative capabilities across diverse domains. These models operate by explicitly modeling data distributions or implicitly generating samples through adversarial or probabilistic frameworks. For example, GANs can generate realistic samples from latent space without relying on distributional assumptions, making them versatile for applications like image synthesis, image translation, and domain adaptation [120]. Compared to other deep generative models, GANs offer notable advantages, including generating higher quality and sharper images than VAEs [42]. Large-scale models such as BigGAN leverage vast computational resources to generate diverse high-resolution images. Transformers, which are known for their self-attention mechanisms, excel in sequential data tasks such as text generation. Flow-based models directly model data distribution, offering efficient sampling and high-fidelity image generation

VAEs are particularly relevant to the development of the LFM due to their ability to efficiently encode and generate complex, high-dimensional data in a structured latent space. They play an important role in enabling the modeling, prediction, and simulation of diverse urban flows by capturing essential spatial and temporal patterns within incomplete datasets. VAEs are probabilistic generative models that encode data into a compressed latent space and decode it to create new, similar data instances. Leveraging neural networks with encoder-decoder architectures enables VAEs to capture essential data features in a simplified latent space, enabling efficient data representation and generation. Regularization during training ensures that the latent space retains desirable properties, supporting the creation of coherent and meaningful outputs [121]. Kingma and Welling [44]) introduced a scalable algorithm for stochastic variational inference, enabling VAEs to train efficiently on large datasets with continuous latent variables. Subsequent research has explored both strengths and vulnerabilities of VAEs. Lu and Chen [45] emphasize adversarial robustness, identifying vulnerabilities in VAEs' latent spaces, such as discontinuities and mismatches with the true data manifold, which can expose them to adversarial attacks. The authors suggest that adversarial training can improve the resilience of generative autoencoders, particularly in applications like communication and compressed sensing, where robust encoding is critical. Meanwhile, Connor et al. [43] address limitations in the latent space structure by proposing the VAE with Learned Latent Structure (VAELLS). Incorporating a learnable manifold model enables VAELLS to refine the latent space, improving data modeling accuracy and enabling class-specific transformations. The model's success in synthetic and real-world datasets demonstrates its potential to better capture complex data patterns.

Concerning this study, while VAEs offer significant advantages in probabilistic modeling and data generation, Autoencoders (AEs) leverages a deterministic architecture, specifically designed to address the spatial, temporal, and multimodal complexities of urban flow data. This approach ensures robust representation and reconstruction of diverse urban dynamics within the UDT framework. One of the strengths of the Blue City Autoencoder (BCA) is its ability to process diverse types of data found in urban environments, including street, flow and satellite data. This versatility is crucial for capturing the full spectrum of urban dynamics. The BCA is trained in an unsupervised manner; it learns to capture the underlying structure and patterns in the data without relying on input and output data.

Recent research highlights the evolution, challenges, and applications of AEs and their advanced variants in DL and generative modeling. Li et al. [122] provide a foundational, comprehensive survey on AEs, emphasizing their evolution, taxonomy, and applications in domains. The authors identify AEs as robust tools for unsupervised learning, particularly for feature extraction and dimensionality reduction, and domain-specific adaptations. However, it also highlights challenges such as limited scalability and interpretability, proposing future integration with advanced DL paradigms as a potential solution.

Pham et al. [123] propose a novel autoencoder inspired by Principal Component Analysis (PCA), designed to improve the organization and control of latent spaces in generative models. By arranging latent dimensions by importance and ensuring statistical independence, the PCA-AE disentangles intrinsic attributes of data without requiring labels. The study demonstrates the PCA-AE’s superiority in disentangling data features compared to other approaches and its applicability to pre-trained GANs. While Pham et al. [123] demonstrate how principles of PCA can be integrated into autoencoder design to enhance latent space organization, Fournier and Aloise [124] investigate PCA's comparative performance of autoencoders and traditional dimensionality reduction techniques, such as PCA, in the context of classification tasks. Specifically, their study evaluates the flexibility and computational efficiency of autoencoders—specifically deep and variational autoencoders—relative to PCA using benchmark image datasets. The findings reveal that while PCA and autoencoders produce comparable classification accuracy when the dimensionality is sufficiently high, PCA is computationally far more efficient, being two orders of magnitude faster than neural network-based autoencoders. This work underscores PCA’s continued relevance as a practical and efficient dimensionality reduction technique, particularly for applications where computational resources are limited.

Crowley [125] examines the foundational role of autoencoders in generative networks, focusing on their utility for signal reconstruction and their contribution to self-supervised learning. The study demonstrates how modifications to the loss function, informed by concepts from information theory, enable autoencoders to discover data categories unsupervised. Additionally, it explores the relationship between autoencoders, VAEs, and GANs, highlighting their complementary roles in modern generative frameworks. This exploration connects the theoretical underpinnings of autoencoders to their practical applications in disentangling latent features, as demonstrated in Pham et al.’s [123] PCA-AE. Expanding on this, Ghosh et al. [126] present an alternative to VAEs by proposing a regularized deterministic autoencoder framework. The study critiques VAEs for their theoretical and practical limitations in generative modeling and offers a simpler, deterministic model with equivalent advantages. By introducing regularization schemes and an ex-post density estimation step, their approach achieves high-quality data generation and disentangled latent spaces, rivaling or outperforming VAEs in applications like image synthesis and molecular modeling.

Overall, these studies illustrate a trajectory of innovation in autoencoder research, moving from foundational surveys and challenges to specialized adaptations that improve latent space organization, generalization, and scalability. They demonstrate how autoencoders, as versatile tools, continue to evolve through refinements in architecture, application domains, and integration with broader generative modeling frameworks.

### 2.4 Foundation models and their pretrained subset

GenAI and FMs are complementary technologies that can be used together to create more sophisticated AI systems capable of generating new data, understanding existing data, and performing various tasks across different domains. FMs represent the broader conceptual category of models that serve as a baseline architecture or framework that can be adapted to diverse tasks through additional training or fine-tuning. As such, they encompass the foundational knowledge and structure that supports various AI tasks across different domains and data modalities. PFMs, a specialized subset of FMs, have already undergone the pre-training phase to build a generalized understanding from extensive and diverse datasets. They are fine-tuned for specific domains or tasks, serving as an efficient and robust starting point and reducing the need for large datasets and computational resources during downstream applications.

Notable examples of PFMs and FMs include Large-scale Language Models (LLMs) like GPT-3 (Generative Pre-trained Transformer 3) [57], BERT (Bidirectional Encoder Representations from Transformers) [46], and Text-To-Text Transfer Transformer (T5) [59], Contrastive Language-Image Pre-training (CLIP) [58], as well as generalist geospatial AI [60], geo-FMs ([61]; [127]; [80]), joint FMs [56], and geographic diverse models [55]. As PFMs, LLMs such as GPT-3, BERT, T5, and CLIP are trained on extensive datasets using self-supervised learning techniques and later fine-tuned for specialized tasks. Generalist geospatial AI and Geo-FMs are FMs tailored to geospatial or geographical data, and their training may or may not involve pre-training as a standalone process. However, when fine-tuned, they would fall into the PFM category. Joint FMs and geographic diverse models are FMs designed to address specific modalities or domains, and they can operate either as FMs or PFMs depending on their implementation and application context.

FMs represent a transformative class of large-scale neural networks, designed to learn general features and representations from vast amounts of data using unsupervised or self-supervised learning techniques [78,79,81]. These models can be adapted to a wide range of downstream AI tasks through methods such as fully sampled fine-tuning, few-shot transfer, zero-shot transfer, and linear probing, enabling applications in novel text, images, and objects [60,62,79].

FMs provide a reasonable and efficient parameter initialization for a wide range of downstream applications [81], including semantic segmentation, content generation, and information retrieval. They can adapt to new domains with minimal task-specific data during fine-tuning or transfer learning stages [56]. For example, the development of multimodal FMs for GeoAI present promising opportunities and exceptional performance in tasks across urban geography, geospatial semantics, and remote sensing [79].

Overall, FMs are powerful and versatile tools, enabling a broad spectrum of AI-driven solutions spanning diverse domains and tasks. Their ability to provide robust pre-training and support specialized adaptation underscores their key role in advancing AI research and applications. Distilled from the aforementioned reviewed studies, Table 1 provides a concise summary of the key attributes distinguishing FMs and PFMs, emphasizing their respective contributions to advancing AI applications.

**Table 1:** Comparison of attributes between foundation models and pre-trained foundation models.

Attributes	Foundation Models	Pretrained Foundation Models
Transfer Learning	Intrinsic to the design of FMs, allowing for adaptability to downstream tasks after sufficient training or fine-tuning.	Exemplifies transfer learning as they are pre-trained and can transfer knowledge to new tasks with minimal additional training.
Resource Efficiency	Applies indirectly, as FMs require substantial resources for pre-training but aim to reduce resource needs during adaptation.	Applies directly, as PFMs reduce computational and data requirements for downstream tasks by leveraging pre-trained knowledge.
Generalization	A key goal of FMs during pre-training is to capture broad patterns and knowledge from diverse datasets.	Explicitly demonstrated by PFMs, which utilize pre-trained knowledge to perform well across various tasks, including unseen or novel domains.
Versatility	Designed to be versatile by serving as a foundational starting point for multiple downstream applications.	Practical versatility is demonstrated by PFMs, enabling rapid adaptation and fine-tuning for specific use cases across diverse domains.

This comparison highlights the theoretical strengths of FMs and the practical advantages of PFMs, illustrating their complementary roles in enabling scalable, versatile, and efficient AI-driven solutions across diverse domains, including urban planning and design.

The insights from the distinctions and capabilities of FMs and PFMs form the foundational basis for the conceptual design of LFMs. Utilizing the generalization, scalability, and versatility of these models, the LFM is designed to address the complexities of urban flows and enhance decision-making processes within UDT frameworks, aligning with the environmental objectives of sustainable smart city planning and design.

### 2.5 Artificial intelligence for sustainable smart city planning, design, and digital twin

As a complex computational model, UDT integrates real-time data from various sources, such as sensors, the Internet of Things (IoT) devices, satellite imagery, and municipal databases. It aims to provide a comprehensive and dynamic representation of urban environments, including infrastructure, buildings, systems, natural resources, and social dynamics. Through the simulation of the behavior and interactions of urban dimensions, UDT enables stakeholders to analyze, visualize, and optimize urban systems, make informed decisions, and test different scenarios for sustainable urban development.

In recent years, there has been a significant increase in the application of AI models and techniques to address the diverse challenges associated with UDT systems, enhancing their capabilities for data-driven planning and design to advance environmental goals. This synergistic integration of AI with UDT has transformed sustainable smart city planning and design, extending the scope of AI-driven UDT systems across various environmental domains. Bibri et al. [11] examine the foundational aspects of AI, AIoT, UDT, urban planning, and environmental sustainability, proposing a conceptual framework for their integrated application in sustainable smart cities. This study highlights the transformative role of AI in advancing UDT systems, enabling the creation of sustainable, resilient, and environmentally conscious urban environments, and shaping the trajectory of sustainable urban development. The environmental domains addressed in this study are mobility flows, transportation optimization, energy efficiency, waste management, and biodiversity conservation. For instance, AI applications, particularly ML and DL, in mobility and transportation have made significant strides. Kamal et al. [128] introduce a DT-Based Deep Reinforcement Learning (DRL) approach for adaptive traffic signal control. This innovative method dynamically adjust traffic signals, reducing delays and enhancing overall traffic efficiency in urban environments. Wu et al. [129] provide a comprehensive classification and analysis of AI and digital twin applications in transportation infrastructure, highlighting their role in predictive maintenance, traffic flow optimization, and resource management. Salunke [24] examines the integration of reinforcement learning-empowered digital twins across various smart city domains, including intelligent transportation systems, energy management, and urban planning. The author underscores the significant potential of combining reinforcement learning algorithms with digital twin technology to optimize traffic flow, alleviate congestion, and improve urban mobility.

From a technical perspective, Zvarikova et al. [23] propose UDT algorithms incorporating ML and DL to ensure accurate urban simulations. Austin et al. [20] demonstrate AI's practical impact in smart city contexts through semantic knowledge representation and ML integration in UDT. Beckett [21] underscores AI's potential to enhance urban design and planning strategies through 3D modeling and spatial cognition algorithms. From a general perspective, Kreuzer et al. [22] reveal that while many instances of integrating AI and DTs employ AI to enhance DTs, there is a noticeable scarcity of sophisticated modeling of the DT itself. Most studies emphasize implementing and testing AI components rather than fostering a robust virtual-to-physical link with the actual systems they mirror. In addition, few studies utilize real-time data to create a physical-to-virtual connection, underscoring a gap in integrating real-world dynamics into DTs effectively.

### 2.6 Generative artificial intelligence and pretrained foundation models for sustainable smart city planning and design and digital twin

GenAI and PFMs are transforming urban planning and design by serving as advanced tools for data-driven decision-making, innovative problem-solving, and enhanced predictive capabilities in sustainable smart cities. By harnessing their robust functions—including analyzing vast datasets, modeling complex urban systems, simulating diverse scenarios, generating actionable insights, synthesizing novel alternatives, and optimizing spatial configurations—GenAI and FMs enable the creation of resilient, efficient, and environmentally sustainable urban environments, aligning urban development practices with the holistic goals of sustainable smart city frameworks.

Bibri [9] examines the transformative role of AI, particularly its generative capabilities, in advancing sustainable smart city planning and design through the utilization of UDTs. The study identifies a significant gap in the literature concerning the integration of GenAI into UDT frameworks as data-driven planning and design systems. The findings highlight GenAI's pivotal role in enhancing data-driven urban planning and design processes via UDTs, revealing its untapped potential in propelling sustainable smart city domains, including energy, mobility, transportation, and infrastructure management. Saranya et al. [51] highlight that Intelligent UDTs integrate real-time analytics, visualization, and spatiotemporal frameworks to derive comprehensive insights from diverse urban data sources, including social and sensor data. However, the reliance on high-dimensional, multi-domain data and challenges related to limited data availability create significant barriers, particularly in generating urban scenarios and design alternatives. GenAI emerges as a promising solution to address these limitations by producing synthetic, high-quality data. The study explores the integration of GenAI with UDT to enhance the management and planning of urban subsystems, such as infrastructure, energy, water, and transportation.

Xu et al. [54] investigate the innovative use of GenAI techniques and UDT to tackle data-related challenges in smart cities. Their review examines how recent advancements in GenAI are transforming smart city applications, focusing on various GenAI models, including GANs, VAEs, transformer-based models, and generative diffusion models. The study highlights GenAI's advanced capabilities to address challenges in UDT applications. These challenges include data augmentation in mobility and transportation management, urban energy systems, and building and infrastructure management; data synthesis and scenario generation in mobility and transportation management, urban analytics, urban energy systems, urban water management, and urban disaster management; as well as 3D city modeling, 3D building generation, 3D street environment, and generative urban design and optimization. Key findings reveal GenAI's potential to substantially aid in these areas, contributing to more sustainable and resilient smart city developments.

Through its ability to model, predict, and generate contextually relevant solutions across diverse urban domains, GenAI empowers planners and designers to address intricate challenges with unprecedented precision and creativity. The outputs of these capabilities are extensive and varied concerning urban planning [9,[27], [28], [29], [30], [31], [32],^84^,^85^] and design (Cheng et al., 2023; [32,[34], [35], [36], [37],^39^,^40^,^77^,^130^]) processes. They include synthetic data for scenario testing, optimization, and simulation processes; innovative design proposals for urban spaces; adaptive spatial plans; scenario simulations for visualizing potential outcomes; virtual environments representing proposed urban developments; predictive models for urban phenomena; customized solutions tailored to specific urban challenges; and virtual urban and architectural design alternatives. Together, these processes and outputs exemplify how GenAI advances the efficiency, sustainability, and resilience of urban systems while providing actionable insights for evidence-based planning and design.

Recent advancements in LLMs, a subset of PFMs, have significantly influenced urban planning and transportation. These models, exemplified by GPT-4 and other domain-specific adaptations, exhibit capabilities such as generating synthetic data, facilitating decision-making, and simulating stakeholder dynamics in urban contexts. Recent research illustrates the application of LLMs in participatory planning, transportation management, and urban design. Zhou et al. [31,32] developed an innovative multi-agent collaboration framework using LLMs for participatory urban planning. The framework simulates planners and diverse resident profiles to create inclusive land-use plans. Using a fishbowl discussion mechanism to improve deliberation efficiency, this model achieves high resident satisfaction and service accessibility, outperforming traditional human-expert approaches in ecological metrics. Jiang et al. [83] introduced UrbanLLM, a fine-tuned LLM designed for complex urban activity planning and management. By decomposing urban queries into manageable sub-tasks and leveraging spatio-temporal AI models, UrbanLLM reduces reliance on human expertise and demonstrates superior performance in addressing urban planning challenges compared to established LLMs like GPT.

In addition, Fu et al. [86] explored the use of ChatGPT in evaluating complex urban planning documents. While ChatGPT aligned with human coders in plan evaluation with 68% similarity, it struggled with domain-specific jargon, indicating its potential as a complementary tool for minimizing human errors rather than replacing human expertise in plan evaluations. Babbar [131] emphasized the potential of specialized LLMs, such as PlanGPT, in addressing urban planning challenges like complex spatial problems and regulatory compliance. These tools streamline the planning process, offering precise, actionable insights to urban planners and setting the foundation for improved productivity and sustainability. Hasan et al. [28] introduced an AI-driven urban planning chatbot that interprets complex legal language and provides actionable recommendations. Utilizing advanced NLP and ML enables the chatbot to promote regulatory compliance and environmental sustainability, reducing human errors in urban planning processes.

In the domain of transportation, for example, Ying et al. [132] evaluated LLMs like GPT-4 and Phi-3-mini in transportation planning. GPT-4 demonstrated higher accuracy and spatial comprehension in Geographical Information System (GIS) and transportation tasks, outperforming Phi-3-mini but highlighting the latter's utility in resource-constrained settings. Challenges related to integrating advanced retrieval-augmented generation techniques were identified for future research. Wang et al. [130] designed an LLM agent framework for personal mobility generation. This framework incorporates real-world urban mobility data, aligning activity generation with individual patterns and motivations through retrieval-augmented strategies. It demonstrated robust performance, marking a significant advance in urban mobility analysis. Yu and McKinley [133] introduced a synthetic participatory method utilizing LLMs to simulate diverse stakeholder inputs for planning shared automated electric mobility systems (SAEMS). A case study in Montreal demonstrated the method's capacity to generate controllable and comprehensive plans, suggesting its potential to revolutionize multi-stakeholder transportation planning. Jin and Ma (Ma, 2024) proposed an LLM-enhanced framework for parking planning during the coexistence of autonomous and human-driven vehicles. This model facilitates efficient evaluation and optimization of parking facilities, achieving notable success rates in testing modules while addressing LLM trustworthiness challenges.

These studies highlight several challenges: understanding domain-specific jargon [86], addressing LLM trustworthiness [87], and integrating advanced generative techniques [132]. Moreover, ensuring scalability and inclusivity in participatory planning frameworks and balancing computational efficiency with model reliability remain significant barriers. Nevertheless, FM and PFMs, especially LLMs, have demonstrated significant potential in urban planning and transportation, addressing complexities ranging from participatory planning to infrastructure optimization. These advancements promise more inclusive, efficient, and adaptive urban environments, although addressing their current limitations is essential for broader adoption and application.

Furthermore, in many real-world applications, particularly those dealing with complex and multidimensional data, FMs play an important role in enhancing the capabilities of GenAI models and, hence, GenAI-driven UDT. FMs boost the performance, accuracy, and adaptability of GenAI models in these contexts by providing rich features and representations learned from vast datasets. Trantas and Pileggi [82] explore the integration of FMs into DT applications, emphasizing the potential to enhance predictive analytics, adaptive learning, and the management of complex datasets. The study highlights the ability of FMs to improve the accuracy and reliability of decision-making processes within DT systems due to their advanced learning capabilities. Using selected ongoing cases, the authors illustrate the practical benefits of this integration, such as more informed and timely decisions derived from comprehensive data analytics and predictive insights. However, they also identify key challenges, including high computational demands, data privacy concerns, and the need for transparency in AI decision-making. The paper positions the integration of FMs and DTs as a transformative step toward advancing AI applications across various domains. In addition, deploying FMs on edge servers leverages their capabilities in distributed and resource-constrained environments. Edge computing's inherent low latency and flexibility enable efficient fine-tuning and inference processes for FMs, which can be dynamically accessed by downstream AI services to support real-time applications [56]. The deployment of FMs on edge servers significantly enhances generative content and DT by leveraging edge computing capabilities to optimize AI-driven processes [56]. This integration improves scalability and responsiveness in urban planning and design tasks, underscoring the importance of FMs in advancing sustainable smart city solutions. In addition, Ali et al. [134] focus on the use of FMs in developing DTs for cyber-physical systems (CPS). The study highlihgts the potential of these models to enhance the functionality and effectiveness of DTs, potentially even allowing them to function as DTs themselves. It also discusses challenges of applying FMs broadly and uses autonomous driving systems as a case example.

Drawing on the reviewed studies, the integration of GenAI and UDT establishes a mutually beneficial relationship that addresses critical challenges in urban planning and design. GenAI, through advanced models, such as GANs, VAEs, and transformers, enhances UDT functionalities by enabling predictive modeling, scenario testing, and generative capabilities tailored to urban flows. FMs adapted to these flows optimize UDT’s ability to analyze complex systems, improve decision-making processes, and enhance predictive accuracy by capturing the interdependencies and dynamics of urban flows. This adaptation enables more effective modeling of spatial-temporal patterns, simulation of urban scenarios, and generating actionable insights to support sustainable urban planning and design. Conversely, UDT, with its high-fidelity and dynamic digital representations of urban environments, serve as a robust platform for testing and operationalizing GenAI-driven solutions tailored to urban challenges. This interplay enables UDT frameworks to leverage GenAI’s computational power while applying its outputs to real-world contexts. Consequently, this makes these frameworks more adaptive and context-aware and ensuring generative insights are seamlessly translated into actionable urban strategies Integrating FMs tailored to urban systems enables UDTs to bridge the gap between advanced generative technologies and actionable urban needs, advancing decision-making processes and fostering long-term environmental sustainability.

### 2.7 Research gaps

Despite the important advancements highlighted in the recent studies, significant gaps persist in research and practice, reflecting the nascent stages of technological convergence. Notably, research on the integration of GenAI and FMs into UDT frameworks remains scant. Current efforts lack a structured theoretical foundation to effectively address complex urban flows and multimodal dynamics, especially concerning modeling and analyzing critical domains, essential for achieving environmental sustainability goals. These multimodal dynamics reflect the constantly changing, interrelated processes, behaviors, and flows across diverse modalities within urban environments. They highlight the inherent complexity, variability, and spatial challenges that span multiple scales and interconnected domains. Existing frameworks often struggle to effectively model or operationalize these intricate dynamics, creating significant obstacles to generating actionable insights for sustainable urban planning and design.

GenAI and FMs hold significant potential to bridge these gaps by adaptively learning, generating, and synthesizing insights from vast, multimodal datasets. UDT frameworks complement these capabilities by simulating, modeling, analyzing, predicting, and optimizing urban dynamics to provide actionable intelligence for urban planning and design. However, addressing the multimodal and spatial complexities of urban systems remains an unmet challenge, emphasizing the need for innovative frameworks to support informed decision-making and advance sustainable urban development. Furthermore, the operationalization of GenAI, FMs, and UDT as a unified system in real-world settings remains critically underexplored, with key challenges related to domain-specific fine-tuning, adaptability, and scalability.

To address these interrelated gaps and limitations, Subsection 7.2 proposes a foundational framework aimed at guiding the integration of GenAI and FMs into UDT systems. Section 5 builds upon this foundation by presenting the LFM architecture, designed to enhance UDT computational and predictive functionalities for enhancing sustainable smart city planning and design. By addressing these gaps and limitations, this research work advances the theoretical and practical dimensions of AI-driven, environmentally sustainable urban development.

### 2.8 A foundational framework for the LFM tailored for UDT within the GSAI framework: A bifocal approach

**Fig. 2:**
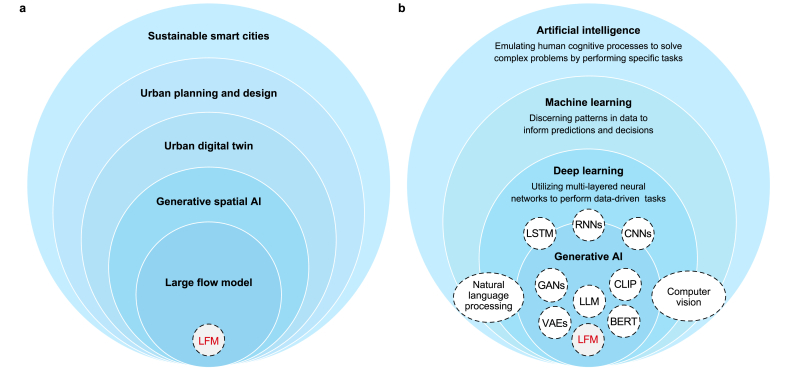
A foundational framework for the large flow model in sustainable smart urban development (a) and Artificial Intelligence and its subfields (b).

The foundational framework (Fig. 2) presented in this section is developed based on insights synthesized from the studies reviewed in the previous section. It offers a comprehensive understanding of AI and its subfields, emphasizing their relevance and significance to UDT as a data-driven planning and design system tailored for sustainable smart cities. This framework seeks to clarify the interconnectedness between these components and their potential for advancing urban planning and design through GenAI-driven UDTs underpinned by FMs. By integrating key theoretical concepts, practical insights, and empirical evidence from the literature, the framework provides a structured approach to tackling spatial challenges associated with UDTs to advance environmental sustainability goals. Its purpose is to enhance UDTs' modeling and simulation capabilities, enabling more informed and efficient decision-making processes for the planning and design of sustainable urban environments.

The LFM serves as a practical endeavor of GenAI and FMs by operationalizing their synergistic and complementary integration into a concrete FM for urban flows. The bifocal approach in Fig. 2 illustrates the nuanced relationship between the LFM and the broader urban context and its connection to urban AI. Specifically, it elucidates how the LFM intersects with the urban landscape, including UDT, urban planning and design, and sustainable smart city development, and integrates with AI and its subfields.

The introduction of the LFM as a novel class of FMs tailored for urban flows underscores the specialized focus of GSAI within the broader realm of urban FMs, including GenAI is a key subset. The LFM is a specialized subset of FMs and a distinct branch of GSAI, designed specifically to model and address the intricate dynamics of urban flows related to the physical and spatial structures of urban environments. This progression from AI to GenAI, then to FMs, and ultimately to LFMs within the GSAI framework represents a continuum of increasing specialization in urban AI. Each step refines the scope and methodologies to tackle the unique challenges inherent in urban planning and design, offering advanced tools for data-driven and predictive urban management.

Furthermore, the LFM shares conceptual and methodological similarities with other GenAI models, such as GANs and VAEs, particularly in its ability to generate new data instances based on learned distributions. However, the LFM distinguishes itself through its specialized focus and purpose. Unlike general-purpose generative models, the LFM is specifically designed to learn and model flow patterns within urban environments. It emphasizes generating and predicting flow sequences, enabling the analysis of dynamic and interrelated urban systems while addressing the unique challenges associated with spatiotemporal data and multimodal urban flows.

Lastly, it is essential to highlight the difference between the LFM and GSAI. GSAI is a specialized domain of GenAI focused on generating spatial data and insights in urban spaces. It encompasses various models and techniques tailored for spatial tasks, such as analyzing and predicting urban flows, analyzing spatial patterns, optimizing urban spaces, and generating spatial configurations. It involves the integration of DL models, geospatial data, and spatial analysis to address complex urban challenges. The LFM, on the other hand, is a specialized type of FMs within the domain of GSAI, specifically designed for analyzing and predicting urban flows. It is trained on large datasets containing unstructured and structured spatial data on the movement and distribution of mobility, goods, energy, waste, materials, and biodiversity flows in urban environments. It leverages techniques from DL and, hence, GenAI models and FMs to capture complex spatiotemporal patterns in flow data, make predictions about future flows, and analyze existing ones. Overall, GSAI is the overarching field that involves various GenAI models and FMs for spatial tasks related to urban planning and design.

Regarding theoretical relevance, the LFM functions as both a foundational framework and a computational tool to address challenges in urban flow modeling within UDT frameworks. Existing urban planning and design theories have struggled to capture the dynamic interrelations of urban flows. Making use of the capabilities of FMs and GenAI, the LFM introduces a novel approach that integrates generative, adaptive, and predictive analytics to model and forecast urban flows. This approach provides a theoretical lens for understanding the interplay between spatial patterns, flow dynamics, and sustainable development objectives. For instance, the LFM‘s ability to complete impartial city data and predict flow sequences challenges traditional assumptions about data dependencies in urban planning and design. Furthermore, the LFM redefines urban flow modeling by capturing spatiotemporal dynamics, interdependencies, and variability across urban systems, extending theoretical models of urban planning and design. These contributions fill critical theoretical gaps in urban flow modeling and establish a robust foundation for exploring GSAI as a paradigm for sustainable urban development. Addressing these gaps enables the LFM to advance the development of sustainable smart cities through improved urban modeling and simulation capabilities.

## 3 Case study: The Blue City Project

This section provides a descriptive account of the Blue City Project, which serves as the overarching case study for this research. Thomas ([135], p. 513) defines cases studies as “analyses of persons, events, decisions, periods, projects, policies, institutions, or other systems that are studied holistically by one or more methods. The case that is the subject of the inquiry will be an instance of a class of phenomena that provides an analytical frame—an object—within which the study is conducted and which the case illuminates and explicates.” In this research work, the case study focuses on analyzing one project—Subproject 8—of the Blue City Initiative. Specifically, as regards the analytical framework, while the focus is on Subproject 8, it is intrinsically linked to other subprojects within the Blue City Initiative, as it relies on multimodal data integration and insights derived from Subprojects 2–6, which cover key urban domains—mobility, goods, energy, materials, waste, and biodiversity. This interconnected framework ensures that the design and development of the LFM for UDT reflects holistic urban dynamics and supports the overarching goals of sustainable smart city planning and design in Lausanne City. This illustrative case study provides a detailed examination of the phenomenon and process within their real-world setting [[136], [137]], where the former entails integrating the LFM into the UDT framework within the Blue City Project and the latter involves its design and ongoing development phases. Together, these aspects provide a comprehensive understanding of the LFM's significance and implications for sustainable urban development. In this context, the case study outlines the specific characteristics, objectives, foundations, functionalities, processes, and intended outcomes of the LFM tailored for UDT. Through descriptive accounts, visual aids, and illustrative examples, it aims to provide a detailed and nuanced understanding of the phenomenon and process, showcasing the LFM’s role and potential impact on sustainable smart urban planning and design in the Blue City Project context.

### 3.1 Overview, progress, and status

The Blue City Project is an innovative and comprehensive initiative designed to transform urban planning and management by developing an advanced UDT framework. Centered in Lausanne City, Switzerland, the project integrates diverse urban data systems to enhance interoperability, streamline data workflows, and promote sustainable urban development. It specifically targets critical urban challenges, such as optimizing transportation networks, improving energy efficiency, reducing resource wastage, enhancing logistical efficiency, and preserving ecological balance. Harnessing the potential of state-of-the-art technologies and fostering collaboration among city planners, engineers, data scientists, policymakers, and industry stakeholders, the Blue City Project aims to create a connected, adaptive, and resilient urban environment that addresses present-day challenges while anticipating future needs.

Since its inception in 2022, the Blue City Project has achieved several significant milestones across its various subprojects. Progress includes the development of an open and secure data platform, advancements in urban mobility optimization, enhanced supply chain logistics, improved waste management systems, and the development of innovative energy management strategies. In addition, the project has made progress in mapping and analyzing material flows to better understand urban resource dynamics. These accomplishments have been driven by the collaborative efforts of city authorities, industry partners, and academic institutions. Looking ahead, the project aims to refine its tools and methodologies, enhance the integration of diverse datasets, tackle emerging urban challenges, address associated risks, and continue advancing sustainable urban development practices.

### 3.2 A Brief Description of Eight Subprojects

**Fig. 3:**
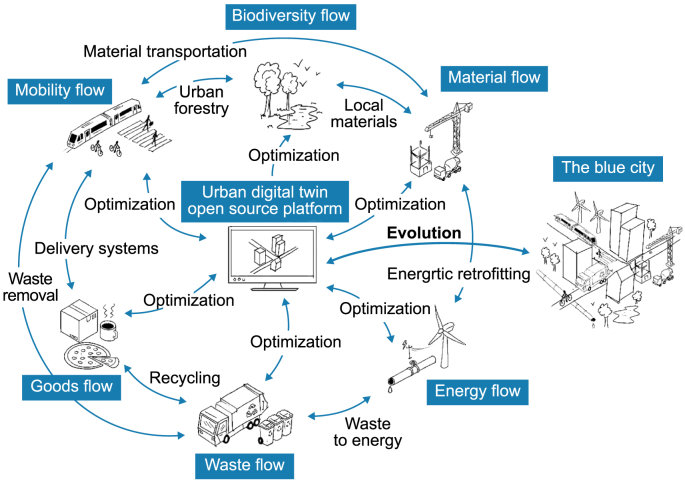
Structure of the Blue city project.

The Blue City Project involves several interconnected subprojects, each focusing on a specific aspect of urban life. These subprojects are designed to work together, harnessing synergies and leveraging collaborative dynamics to address a range of urban challenges (Fig. 3). Examining the relationships between these subprojects reveals how the integrated approach enhances the practical relevance and overall effectiveness of the project. This interconnected framework ensures that the insights and innovations from each subproject contribute to the broader goals of creating sustainable smart urban environments.

The subprojects of the Blue City initiative are described below, with a focus on their aim, achievements, and current results:

**Subproject 1: Urban Data Platform** aims to ensure compatibility and interoperability of diverse urban data systems. Developing the Data Mesh Platform using Kafka enables real-time data streams, seamless data integration, and secure data exchanges. Embedded in the Data Mesh Platform is a robust Security System designed to address data privacy and security concerns, ensuring that data flows are protected during real-time exchanges. The Data Storage System complements these capabilities by serving as a centralized repository for managing static file sharing. It securely stores essential datasets from Lausanne City and ensures their availability for reliable retrieval and analysis by researchers across various subprojects. Together, these integrated systems establish a robust data collection, integration, and analysis infrastructure. The project highlights the importance of effectively deploying a scalable data architecture, embedded security mechanisms, and repositories, which serve as the backbone for other subprojects by providing the necessary resources for their operations.

**Subproject 2: Urban Mobility** focuses on developing an accurate, fast travel time engine and accessibility measures to enhance urban mobility planning. Significant achievements include enhancing the Open Source Routing Machine (OSRM) for high-accuracy travel time calculations and multi-scale accessibility measures. These advancements have introduced innovative multi-scale accessibility measures, allowing detailed evaluation of infrastructure changes, aiding strategic urban mobility planning. The results so far indicate improved tools that urban planners can use to make informed decisions about transportation systems.

**Subproject 3: Biodiversity** aims to map and understand urban biodiversity to promote conservation efforts. The integration of georeferenced biodiversity data and the development of an interactive mapping tool have been key achievements. These tools visualize urban ecological networks, clearly showing how urban development impacts biodiversity. The project has introduced methods to assess urban wildlife patterns, and the results have aided in identifying critical areas for conservation.

**Subproject 4: Waste Management** is dedicated to analyzing waste flows and developing resource-efficient strategies. The project has developed a GIS platform for visualizing waste generation and flow and initiated predictive models to forecast future waste production. These achievements have significantly enhanced the understanding of urban waste management systems. The project has set a new standard for data-driven waste management research, and the results have provided insights into optimizing waste collection and recycling processes.

**Subproject 5: Energy Management** aims to create a digital representation of the energy system to optimize energy flows. Developing an energy system model linking district- and national-level systems has been significant. This model allows for detailed visualization and optimization of energy consumption within Lausanne. The project has leveraged GIS technology to provide insights into energy distribution, and the results have supported strategic energy planning and sustainability efforts.

**Subproject 6: Material Flows** focuses on mapping material usage, composition, and distribution within the city. Key achievements include comprehensive mapping of construction materials and developing GIS tools for visualizing material stocks. These tools support sustainable building practices and resource management by providing detailed insights into material flows. The project has proposed frameworks for incorporating circular economy principles into urban planning, and the results have facilitated better decision-making in construction and resource allocation.

**Subproject 7: Supply Chain Optimization** aims to optimize urban logistics and enhance delivery services. Developing a Vehicle Routing Problem (VRP) solver using Branch-Price-and-Cut algorithms has been a significant achievement. This tool helps SwissPost, an industrial partner, determine the optimal fleet composition and distribution center locations, reducing costs and improving service quality. The project's collaboration with the City of Lausanne and using data from Sparrows on SwissPost delivery vans have been instrumental in this achievement. The project has contributed to logistics optimization research, and the results have demonstrated considerable improvements in urban delivery efficiency.

**Subproject 8: The Blue City – Multidimensional, AI-assisted Platform for Proactive City Planning** represents the culmination of the Blue City Project, building on the integrative foundation established by Subproject 1. It is focused on creating a responsive, open-source tool for proactive city planning by synthesizing aggregated data and knowledge from the ancillary subprojects. This project combines design and data science with AI and DL technologies, laying the foundation for collective human-machine intelligence. This intelligence will empower city governance and citizens alike, offering deeper insights into urban systems and enabling the exploration of innovative planning possibilities. The ultimate goal of Subproject 8 is to develop a new type of multidimensional, openly accessible urban simulation platform. This platform will support proactive decision-making, promote urban well-being, and foster sustainable urban solutions. The project aims to transform strategic planning and innovation in sustainable urban management by enhancing the ability to understand and anticipate urban changes.

As part of Subproject 8, significant progress has been made in designing and developing the LFM, equipped with GenAI capabilities. This advanced model is essential for simulating and predicting various urban flows while integrating insights from all contributing subprojects. At its current stage, the project has successfully completed the design phase of the LFM and is actively progressing through its ongoing development, which serves as the focal point of this study. The LFM is poised to be a transformative tool for understanding complex urban dynamics and shaping the future of city planning and management.

### 3.3 Synergies and relationships to the urban digital twin framework

Each subproject within the Blue City Project is designed to contribute to and benefit from the overarching UDT framework. As detailed earlier, Subproject 1 provides the essential infrastructure and data integration capabilities required for all other subprojects, which is essential for developing the UDT platform.

Subprojects 2–7 function as specialized UDTs focused on specific urban flows. The synergies among these subprojects enhance the overall functionality and effectiveness of the Blue City Project in terms of the underlying UDT framework planned for development, fostering a holistic and integrated approach to urban management. For instance, Subproject 4: Waste Management and Subproject 6: Material Flows complement each other by analyzing materials' lifecycle and eventual disposal. Insights from waste management inform sustainable building practices and material recycling strategies, while material flow data helps optimize waste reduction initiatives. Subproject 2: Urban Mobility and Subproject 5: Energy Management interact by examining how transportation systems influence energy consumption and vice versa, leading to more efficient and sustainable urban mobility solutions. Subproject 3: Biodiversity also interrelates with others by assessing how urban development (informed by mobility and construction data) affects ecological networks, promoting biodiversity-friendly urban planning and design. Moreover, Subproject 7: Supply Chain Optimization integrates findings from urban mobility and material flows to enhance the efficiency of logistics and reduce environmental impact.

### 3.4 Conception of subprojects as specialized urban digital twins

The subprojects of the Blue City Project, except for Subproject 1 and Subproject 8, are conceptualized as individual UDT platforms, each focusing on specific facets of urban life. These subprojects intend to create detailed digital models of urban flows within their respective domains, leveraging the foundational platform for analysis and optimization. Their purpose is to deliver actionable insights supporting proactive urban planning and design and informed decision-making. Subproject 1 contributes to integrating these individual UDT platforms, fostering collaboration among various stakeholders and ensuring seamless connections across various domains. It is intended to enable the creation of a unified and interconnected digital representation of the entire City of Lausanne, which serves as the backbone for comprehensive citywide analysis and optimization. Building upon this integrated platform, Subproject 8 focuses on developing a multidimensional, AI-assisted tool for proactive city planning and management. By synthesizing the collective data and insights from all subprojects, Subproject 8 enables innovative and sustainable urban development solutions, setting the stage for a more responsive, efficient, and resilient urban future.

### 3.5 Evolving the open data platform into a comprehensive UDT system

Integrating the Data Mesh Platform, Security System, and Data Storage System forms the foundation for developing the UDT platform by combining dynamic and static data flows, enabling accurate simulations, advanced analytics, stakeholder collaboration, and informed decision-making pertaining to urban planning and management. This ensures the UDT platform effectively mirrors the complexities of the urban environment while maintaining data integrity and security. In the context of Subproject 8, the UDT platform requires real-time simulation, modeling, and predictive analytics to replicate the behavior and dynamics of the physical environment it represents. The open data platform plays a critical enabling role by providing the data infrastructure necessary for the UDT platform. To transform the open data platform into a functional UDT platform, additional components and processes need to be incorporated, extending beyond mere data storage and accessibility:

• Real-time data integration to ensure continuous and up-to-date monitoring of urban systems.
• Dynamic modeling and predictive simulations to reflect real-world behaviors and project future scenarios.
• AI and advanced analytics for generating deeper insights and supporting complex decision-making processes.
• Interactive 3D visualization tools to engage users and explore urban scenarios effectively.
• Application Programing Interfaces (API) connectivity to integrate with other urban systems and foster interoperability.
• A continuous feedback loop to refine decision-making processes through iterative improvements.
• Scenario testing and optimization to support proactive urban planning and resource management.

Incorporating these components enables the open data platform to evolve into a comprehensive, interactive, and real-time UDT platform. This system can become a powerful enabler for smarter, more sustainable urban planning and management, equipping cities to proactively address challenges, optimize resource allocation, and enhance operational efficiency. While this enhanced UDT platform aligns closely with the overarching goals of Subproject 8, the latter extends beyond platform development to integrate advanced AI-assisted modeling, such as the LFM, and to foster collective human-machine intelligence for proactive urban planning and innovation.

### 3.6 Justification for the Study’s setting and sample

The Blue City Project in Lausanne City is an ideal setting for this study due to its multifaceted urban environment, characterized by integrating diverse urban flows, active engagement with sustainability initiatives, and access to a wide range of high-quality datasets across multiple domains. This project exemplifies a comprehensive sustainable smart city initiative, encompassing critical urban domains. These characteristics establish it as a strategic and dynamic testbed for the LFM, enabling the convergence of advanced data-driven methodologies, namely GenAI, FMs, and UDT. Moreover, the project fosters collaboration among city planners, designers, engineers, data scientists, industrial partners, and policymakers, providing both a rich dataset ecosystem and a collaborative environment for addressing real-world challenges. Lausanne City’s strong commitment to sustainability and innovation aligns well with the LFM’s goals of advancing data-driven decision-making for sustainable smart urban planning and design, ensuring the study’s practical relevance and theoretical significance.

The sample for the LFM’s design and ongoing development phases, detailed in the next section, is derived from data generated and handled by subprojects 2–7 within the Blue City Project. These subprojects encompass key urban flows, including mobility patterns, goods transportation, energy consumption, waste management, biodiversity metrics, and material usage. These datasets were carefully selected to align with the LFM’s objectives of modeling and integrating diverse urban flows. Harnessing multimodal datasets across various urban flows enables the LFM to establish a strong foundation for addressing complex and interconnected urban dynamics, facilitating its scalability and adaptability across diverse urban contexts.

In addition, Lausanne City’s ongoing urban development initiatives and integration of advanced technological solutions into urban planning and design make it an exemplary setting for evaluating the LFM’s ability to tackle spatial challenges and generate actionable intelligence. The interconnected nature of the subprojects and their emphasis on urban flows ensure that the LFM undergoes rigorous testing in a real-world, multifaceted urban context. This setting provides an opportunity to validate the LFM’s potential to enhance urban resilience and sustainability, offering theoretical advancements and practical solutions for sustainable smart city development.

## 4 Research methodology: Design and Development phases

This study introduces a pioneering FM for urban flows, developed with GenAI capabilities, specifically tailored for integration into UDT frameworks. To reiterate, the Blue City Project in Lausanne City serves as a strategic and dynamic testing ground for the design, development, subsequent implementation, and validation of the model’s potential to address spatial challenges and provide actionable intelligence for urban planning and design. In this study, the research methodology adopts a phased approach comprising a completed design and ongoing development phases.

The design phase, which is the primary focus of this study, outlines the conceptual design of the LFM, aligning it with the specific requirements of urban flow modeling in complex and dynamic environments. The development phase, currently underway with key milestones projected for 2025, builds upon this design, refining the model through iterative prototyping, data integration, and algorithmic enhancements. Together, these phases ensure that the LFM evolves into a functional, scalable solution that addresses multimodal challenges in urban systems. The following two subsections detail the methodology for each phase.

### 4.1 Conceptualizing and planning the large flow model

**Fig. 4:**
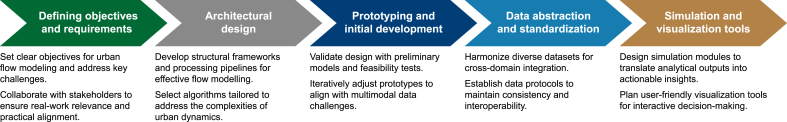
A flow diagram outlining the step-by-step process of the conceptual design of the large flow model.

The design phase lays the groundwork for the LFM, focusing conceptualizing and planning of its architecture, data handling strategies, and simulation capabilities to align it with the specific needs of urban flow modeling in complex and dynamic environments. This phase follows a structured progression (Fig. 4), comprising the following components:

#### 4.1.1 Defining objectives and requirements

The design phase begins with a focused effort to set clear objectives and requirements, forming the foundation for the LFM's development. This involves precisely defining the urban flows to be modeled and identifying specific challenges to address. Active stakeholder collaboration ensures that the LFM's design reflects real-world needs and priorities. By jointly defining functional and technical requirements, this phase ensures that the model aligns with the practical demands of urban planning and design in the Blue City Project.

#### 4.1.2 Architectural design

Once objectives are defined, the architectural design outlines the model's structural framework. This involves developing data flows, storage solutions, and processing pipelines, alongside selecting appropriate algorithms, such as ML and DL models, statistical methods, and optimization techniques, to address the complexities of urban flow modeling. A custom BCA was conceptualized as a key component, with an encoder-decoder structure tailored to handle high-dimensional spatial-temporal data.

#### 4.1.3 Prototyping and initial development

With the architecture defined, this phase validates the LFM’s conceptual design. This step involves creating preliminary models and diagrams to visualize the LFM’s structure and simulate its operation in controlled scenarios. These prototypes serve as a feasibility test, providing critical insights into potential challenges and opportunities. For example, the prototypes assess the model's capability to handle multimodal datasets and its initial performance in representing urban flows. Adjustments are made iteratively to ensure that the design phase results in a robust and adaptable framework for subsequent development.

#### 4.1.4 Data abstraction and standardization

Data abstraction and standardization are important in seamlessly integrating diverse datasets into the LFM. Abstraction layers are designed to harmonize data inputs and outputs, creating a unified structure that accommodates varied urban flow types. In addition, data protocols and standards are developed to ensure compatibility across heterogeneous datasets, fostering interoperability and coherence. This step is critical for maintaining consistency and reliability in the LFM's analytical processes, equipping the model to effectively process and analyze complex urban datasets and enhancing its predictive and modeling capabilities.

#### 4.1.5 Simulation and visualization tools design

The design phase focuses on conceptualizing and planning the simulation modules and visualization tools necessary to translate the LFM's analytical outputs into actionable insights. This involves outlining their functionality, purpose, and role. Simulation modules are conceptually designed to enable scenario testing, providing a framework for representing and exploring potential urban dynamics (e.g., mobility flow variations, energy demand fluctuations). The design ensures these modules align with the objectives of the LFM and the needs of stakeholders. Visualization tools are planned as intuitive, user-friendly interfaces for representing urban dynamics; these tools aim to ensure that stakeholders can later interact with the LFM’s outputs effectively. They facilitate clarity and decision-making by providing easily interpretable representations of the model's analyses.

The design phase culminated in developing a comprehensive blueprint for the LFM, aligning technical innovations with the objectives of the Blue City Project. This structured approach sets the stage for the ongoing development phase, which builds upon the design phase to refine and implement the LFM. Recognizing the evolving nature of urban environments, the design prioritized scalability and adaptability, allowing the LFM to incorporate new data types and expand its application to other urban contexts. This flexibility ensures the model remains relevant and robust as urban challenges and data sources evolve.

### 4.2 Ongoing development phase: constructing and refining the large flow model

**Fig. 5:**
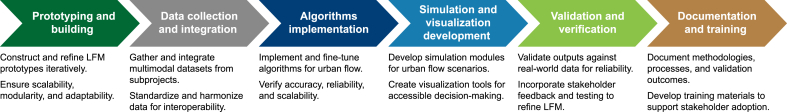
A flow diagram outlining the step-by-step process of the ongoing development of the large flow model.

The ongoing development phase focuses on transforming the LFM’s conceptual design into a functional and operational model. This phase ensures the LFM evolves into a robust tool for urban flow modeling and analysis by integrating outcomes from the design phase with real-world data and insights from the Blue City Project. The process follows a structured progression (Fig. 5), comprising the following components:

#### 4.2.1 Prototyping and building

This stage involves constructing and refining initial prototypes of the LFM, guided by the detailed blueprint developed in the design phase. Developers test the feasibility and functionality of preliminary versions of the model, iteratively refining and improving them based on performance feedback and stakeholder insights. A key focus is scalability and flexibility planning, which ensures the model’s modular architecture, optimized data handling, and ability to generalize across diverse urban contexts. These efforts prioritize adaptability and robustness, enabling the LFM to address emerging challenges in both localized and broader urban scenarios effectively.

#### 4.2.2 Data collection and integration

A critical aspect of the development phase is gathering and integrating diverse datasets from subprojects 2–7 of the Blue City Project. These datasets include mobility patterns, goods transportation, energy consumption, waste management, biodiversity metrics, and material usage. The datasets are harmonized through data abstraction and standardization processes to ensure interoperability and consistency across formats, temporal resolutions, and spatial scales. This integrated framework enables the LFM to process and analyze multimodal urban flows efficiently, forming the backbone of its predictive and analytical capabilities.

#### 4.2.3 Algorithm implementation

The coding and integration of algorithms identified during the design phase are central to this component. Developers ensure seamless interaction between algorithms and datasets, conducting rigorous testing to verify accuracy, reliability, and scalability. Algorithms are fine-tuned to optimize the LFM’s ability to simulate and predict urban flows under various conditions, ensuring robust performance across dynamic urban scenarios.

#### 4.2.4 Simulation and visualization development

In the development phase, the focus shifts from planning to practical implementation and refinement of the simulation and visualization tools. Simulation modules are actively developed to model specific, complex urban scenarios, such as traffic flow optimization, energy distribution planning, or waste management strategies. These modules are programmed and iteratively refined to deliver actionable insights grounded in real-world data. Concurrently, visualization tools are built to translate the LFM's outputs into interactive, user-friendly formats. These tools enhance stakeholder understanding by bridging the gap between complex analytics and evidence-based decision-making, providing accessible visual representations of urban dynamics and predictions. Worth noting is that while the design phase conceptualized the functionality and purpose of simulation and visualization tools, the development phase emphasizes their usability and alignment with stakeholder needs, improving the LFM’s interpretability and applicability in real-world contexts.

#### 4.2.5 Validation and verification

Validation ensures the LFM’s reliability and accuracy by testing its outputs against real-world data from the Blue City Project. Initial validation assesses on the model’s ability to produce realistic and actionable insights, while verification confirms that all components function cohesively. Stakeholder feedback and testing results guide iterative refinements, ensuring the LFM’s adaptability to varying urban contexts.

#### 4.2.6 Documentation and training

Comprehensive documentation supports the operationalization of the LFM, detailing the development process, algorithms, data integration methods, and validation outcomes. Moreover, training materials are prepared to familiarize stakeholders with the LFM’s functionalities and applications. These materials empower users to leverage the model effectively, promoting its adoption in urban planning and design practices.

The ongoing development phase builds upon the completed design phase by constructing and refining the LFM through iterative testing and feedback. This phase ensures that the LFM evolves into a reliable and scalable model for urban flow analysis by integrating diverse urban datasets, developing advanced algorithms, and creating simulation and visualization tools. These efforts contribute to the Blue City Project's objectives of fostering sustainable, data-driven urban planning and design solutions.

## 5 Results: Introducing the pioneering LFM as part of subproject 8 of the Blue City Initiative

This section presents the results and progress of the LFM within the Blue City Project. It focuses on the LFM's design, ongoing development, and its application in modeling and optimizing urban flows to address critical urban challenges. It details the completion of the LFM’s design phase providing a solid conceptual foundation and outlines the ongoing development efforts. The LFM culminates in a comprehensive framework by integrating city flow data, structures, and cutting-edge technologies. These efforts are guided by the goal of creating a functional and scalable tool for urban flow modeling, with the initial prototype targeted for release in 2026. The section also highlights how the LFM is being tested and refined through real-world data from the Blue City subprojects, demonstrating its potential to enhance sustainable urban planning and evidence-based decision-making.

### 5.1 Goal and objectives

**Fig. 6:**
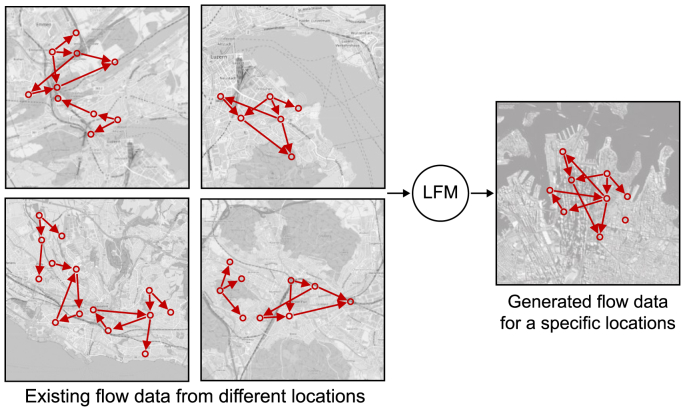
Goal of the large flow model.

The LFM stands at the forefront of urban planning and design innovation, as it harnesses the power of FMs and GenAI to transform how we conceptualize and manage urban environments. It combines techniques from GenAI and FMs with spatial data to generate new insights and predictions about physical spaces and structural flows. Its goal is to learn meaningful representations of existing city flow data from different locations to generate new flow data for specific locations (Fig. 6).

Within this overarching goal, the LFM pursues the following specific objectives:

(1) Completing impartial city data: The incomplete and biased nature of urban data has been a persistent issue. The LFM can assist in filling the gaps in urban data, providing comprehensive, unbiased information about cities.
(2) Estimating flow data for new locations: Understanding and predicting the movement of people, vehicles, and resources is crucial for urban planning, logistics, and disaster management. The LFM can contribute to more accurate flow data estimations, even in locations lacking historical flow data.
(3) Forecasting the evolution of flow data: As cities and spaces evolve, the dynamics of flows change. The LFM has the potential to predict how these flows will evolve in time, supporting informed decision-making and proactive planning.
(4) Holistic urban understanding: Traditional urban data analysis methods focus on specific areas or aspects of urban life, resulting in a fragmented understanding of the city's dynamics and interactions between different components. The LFM combines diverse datasets and analytical techniques to provide a holistic understanding of urban dynamics and their interconnections, enabling planners to consider the broader context and implications of their decisions.

### 5.2 The Blue City large flow model

#### 5.2.1 LFM pretraining

**Fig. 7:**
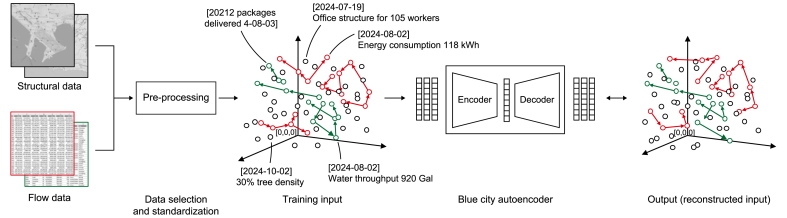
Pretraining the large flow model.

The construction of the Blue City LFM involves several steps (Fig. 7). The process initiates with collecting structural and dynamic flow data. These data then undergo a step of data selection and standardization, followed by preparation of training input, including the incorporation of input embeddings and positional encoding. These refined data are then used to pre-train a foundational model capable of discerning complex urban flow patterns using the BCA.

#### 5.2.2 Fine tuning

**Fig. 8:**
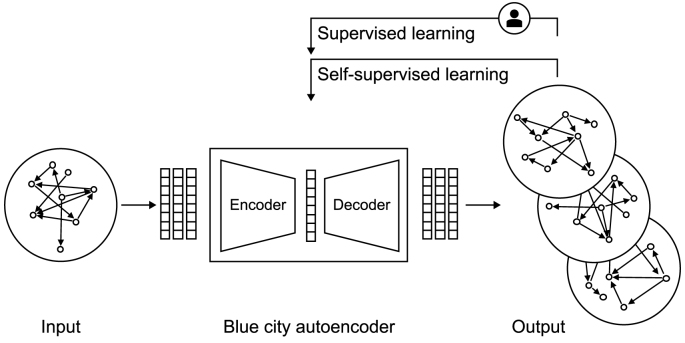
Fine-tuning the large flow model.

The LFM uses supervised and self-supervised learning techniques to fine-tune and optimize the model for specific downstream applications (Fig. 8).

Supervised learning involves training the model on labeled data, where it learns to map input features to target outputs, enabling it to make predictions based on new input data. Furthermore, the supervised learning component of the LFM will benefit from the guidance of Blue City domain specialists in various urban flows. These experts will provide supervision by validating and annotating datasets, ensuring the models align with real-world patterns and challenges.

On the other hand, self-supervised learning leverages unlabeled data to generate auxiliary tasks, allowing the model to learn useful representations and patterns of the input data without requiring extensive manual annotation. This approach uses pretext tasks to create pseudo-labels from the data, enabling the model to learn the underlying structures and dependencies. For example, the LFM might predict missing segments in time-series traffic data or reconstruct energy flow distributions from incomplete records, using contrastive learning or masked autoencoders. Self-supervised learning enhances the model's ability to generalize across diverse datasets and urban contexts by focusing on tasks like temporal forecasting or spatial interpolation. Overall, the dual approach empowers the LFM to adapt and evolve according to the unique requirements of various urban planning and design tasks, ensuring its versatility and effectiveness across different application domains.

#### 5.2.3 Stimuli and downstream applications

**Fig. 9:**
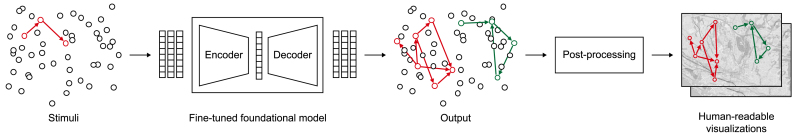
Inference: Downstream application triggered by stimuli.

The LFM uses the fine-tuning technique to generate outputs based on specific inputs (“stimuli”) (Fig. 9). It applies the learned knowledge and patterns acquired during the model’s fine-tuning phase to respond with new and unique content. Through post-processing and visualization techniques, the output is rendered human-readable.

The introduction of FM for urban flows in the context of GSAI represents a significant shift in urban modeling. With its ability to provide a more accurate, comprehensive, and timely picture of urban dynamics, the LFM provides a comprehensive view of urban ecosystems, surpassing the capabilities of traditional data collection methods. This enables stakeholders to construct a complete and nuanced picture of urban landscapes, capturing previously overlooked or inaccessible aspects. This approach considers the interplay between different urban flows and their cumulative impact, providing a holistic understanding of how cities function.

The LFM aligns closely with the objectives of UDT initiatives, which offer urban planners and designers’ novel insights into the impacts of various interventions and strategies. UDT can enhance data-driven decision-making by integrating an LFM into their frameworks, fostering more resilient and efficient urban development pathways. The LFM holds significant promise for infrastructure planning, policy implementation, and addressing social trends by providing detailed insights into urban flow patterns and their spatial-temporal dynamics. As sustainable smart cities evolve, incorporating the LFM into UDT can transform how cities are planned, developed, and managed in the future.

More recently, research has begun to explore the integration of FMs into UDT applications, highlighting their potential to enhance predictive analytics, adaptive learning, and the management of complex datasets. This integration underscores their ability to improve decision-making accuracy and reliability within UDT systems by leveraging advanced learning capabilities. As part of this practical endeavor, this study demonstrates the benefits of this integration, such as enabling more informed and timely decisions through comprehensive multimodal data analytics and predictive insights. It positions the convergence of FMs and UDT as a transformative advancement in the application of AI for urban planning and design.

### 5.3 The core elements of the large flow model

**Fig. 10:**
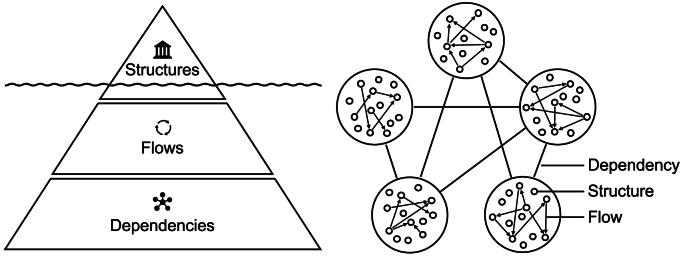
Structures, flows, and dependencies as core terminology underpinning the large flow model.

This subsection presents the foundational components of the LFM, emphasizing how it processes diverse urban datasets and integrates advanced modeling techniques. To ground this discussion, it is important to clarify the primary terminology—structures, flows, and dependencies—that underpins the model’s conceptual design. Fig. 10 provides a simplified representation of the core concepts driving the LFM. Structures represent the physical and visible objects of the urban environment. Flows depict the movement of people, goods, energy, waste, materials and information between these structures, reflecting the dynamic nature of urban systems. Dependencies highlight the interactions and influences between structures and flows, illustrating the cascading effects they can have on each other. This terminology underpins the LFM's ability to effectively model complex urban systems and predict interrelated urban dynamics.

The LFM is built upon a foundation of data collection and aggregation, data abstraction and standardization, and the BCA for DL model training.

#### 5.3.1 Data collection and aggregation

The first step in the LFM process is collecting and aggregating diverse data types that capture the various aspects of urban life. These data form the basis for all subsequent analysis and modeling in the LFM. The project utilizes diverse data sources to inform and train the LFM. Examples of these data sources include waste flow data from a large municipal waste management service, energy flow data from a major regional energy provider, traffic data from a national roads office and open mapping platforms, and materials flow data from construction and recycling databases managed by urban planning authorities. These sources provide context-specific data to enable the modeling of urban dynamics. The datasets collected from the Blue City subprojects serve as the foundation for the ongoing development efforts. These data, categorized into three main types—flow data, structural data, and flow descriptors—are being integrated and processed to validate the LFM's ability to model complex urban flows, providing real-world feedback for iterative refinement.

##### 5.3.1.1 Flow data

As vital components of city life, Flow Data are crucial in understanding the dynamic aspects of urban environments. In the Blue City Project, these data are sourced using advanced tools, systems, or methods, in collaboration with industrial partners and Lausanne City.

• Flow of people: Data on how people move around the city are collected through IoT devices, mobile phone signals, public transportation systems, and pedestrian counters. These data help understand commuting patterns, pedestrian traffic, and population density fluctuations throughout the city.
• Flow of vehicles: Information about vehicular flow, congestion points, and overall traffic patterns is gathered from traffic cameras, road sensors, and vehicle GPS data. These data are instrumental in understanding and managing traffic flows and transportation networks.
• Flow of goods: The movement of goods within the city is tracked through GPS-enabled transportation vehicles and freight management systems. These data provide insights into delivery routes, logistics hubs, and supply chain networks, which are critical for efficient city logistics.
• Flow of energy: Data on the distribution and consumption of electricity, water, and gas are collected from smart meters and utility monitoring systems. These data are key to managing urban energy and utilities infrastructure effectively.
• Flow of waste: Waste collection routes, recycling centers, and landfill activities are monitored through waste management systems and sensors on collection vehicles, providing important data for efficient waste management in the city.
• Flow of biodiversity and nature: These data track the presence and movement of wildlife within urban areas, as well as the distribution of green spaces like parks, gardens, and natural reserves. Techniques such as wildlife cameras, environmental DNA sampling, and biodiversity surveys are utilized. These data help understand the interactions between urban development and natural ecosystems, aiding in creating sustainable urban landscapes that support biomediation and biodiversity.
  • Flow of Materials: This includes tracking the inflow and outflow of construction materials within the city, alongside data on urban mining potentials for promoting circular construction practices. Sources include construction project logs, tracking of material shipments, and assessments of buildings and infrastructure for recyclable materials. These data help manage resources efficiently and foster sustainable construction practices by maximizing the reuse and recycling of building materials within the city.

##### 5.3.1.2 Structural data

Structural Data pertain to the city's physical infrastructure and provide the static framework upon which the dynamic flows interact. These data type include detailed information about the city's built environment:

• Road networks: Detailed information about the city's road networks, including types of roads, lane structures, intersections, and traffic signals, is sourced from mapping services like OpenStreetMap and government GIS databases. These data are essential for understanding the city's transportation infrastructure.
• Buildings and land use: Data on buildings (e.g., residential, commercial, or industrial), including their types, sizes, usage, and zoning information, are obtained from city planning departments and satellite imagery. These data help understand the spatial layout and functionality of different urban areas.
• Public spaces: Information about parks, plazas, and open areas, including their sizes, locations, and amenities, is crucial in understanding the city's public spaces and their role in urban life.
• Utility infrastructure: The location and capacity of utility infrastructure, such as water pipes, electrical grids, and telecommunications networks, are mapped to understand the city's essential services and their distribution.
• Topographical Features: Detailed information about the city's topography, including elevation levels, slope gradients, and geological features, is collected through GIS, satellite imagery, digital elevation models (DEMs), and Light Detection and Ranging (LiDAR) data.

##### 5.3.1.3 Flow descriptors

Flow Descriptors are metadata elements that enrich the Flow and Structural Data, providing additional context and aiding in categorizing and understanding various flow types. These descriptors include temporal information, spatial descriptors, categorical tags, and qualitative descriptors:

• Temporal information: Data are time-stamped to indicate when specific flows occur, helping to identify patterns and trends over time.
• Spatial descriptors: Geographic coordinates and area descriptions provide spatial context to the flows, allowing for a detailed understanding of how different parts of the city are connected and interact.
• Categorical tags: Keywords and labels categorize data into different flows, such as residential versus commercial traffic or renewable versus non-renewable energy sources, offering a clearer understanding of the data.
• Qualitative descriptors: Descriptive information provides qualitative insights into the flows, such as congestion levels in traffic data or pedestrian friendliness in urban design.

#### 5.3.2 Data abstraction and standardization

**Fig. 11:**
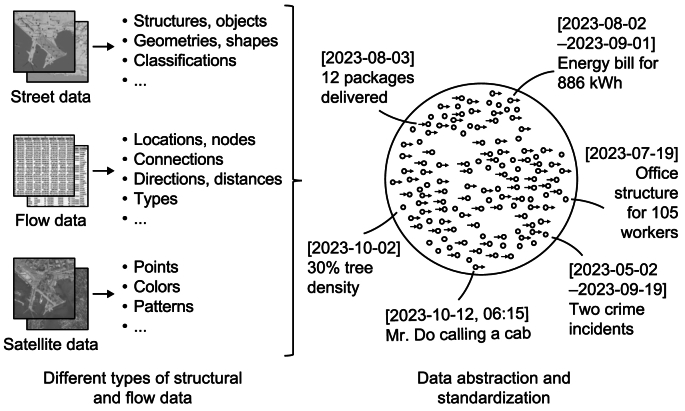
Data selection, abstraction, and standardization.

Once the diverse range of data is collected and aggregated, the next step is to prepare these data for pretraining the LFM. This stage involves two primary processes (Fig. 11): data abstraction and standardization, which ensure that the data are in a format that is compatible with analytical tools and conducive to effective analysis. The abstraction and standardization processes described here build on the data integration steps, where diverse datasets from the Blue City subprojects are harmonized into a consistent format. These ongoing efforts enable the LFM to handle multimodal data and generate meaningful predictions.

##### 5.3.2.1 Data abstraction

In data abstraction, complex and heterogeneous data are converted into a uniform format that is suitable for analysis. Given the varied nature of urban data sources, this step is critical for harmonizing the different data types into a cohesive dataset.

• Format harmonization: The first aspect of data abstraction involves converting data into a consistent format. Urban data come in various formats, such as text, numbers, and dates. Format harmonization ensures that all these data types are standardized, making them compatible for combined analysis.
• Data integration: Another key aspect of data abstraction is data integration from multiple sources. This process involves aligning data from different systems to create a unified dataset.
• Aggregation and disaggregation: Data are aggregated to a higher level or disaggregated to a more detailed level. For example, individual traffic data points can be aggregated into hourly trends, or regional data could be broken down into insights at the neighborhood level.
• Handling anomalies and Outliers: Identifying and addressing data anomalies and outliers is important to data abstraction. Anomalies and outliers can skew analysis results, so filtering out extreme values or apply statistical methods to adjust the data accordingly is essential.

##### 5.3.2.2 Data standardization

Standardization data are prepared to train the LFMs effectively. This step ensures that the data are in a state that allows for accurate and reliable modeling.

• Normalization and scaling: One of the first steps in standardization is to adjust the range of data values to be on a similar scale. This normalization or scaling process—using techniques such as min-max scaling or z-score normalization—prevents features with larger scales from dominating the model's learning process.
• Handling missing data: Addressing gaps in the data is another critical aspect of standardization. Missing data can occur for various reasons, such as sensor malfunctions or incomplete records. Techniques to handle missing data include imputation (filling in missing values), using indicators for missing data, or omitting records with missing values altogether.
• Encoding categorical variables: Urban data often include categorical variables, such as city names or types of roads. These variables are encoded numerically using one-hot encoding or label encoding methods.
• Feature engineering: Creating new features or modifying existing ones can enhance the model's ability to capture important patterns and relationships in the data. Feature engineering involves combining features, creating ratios, or deriving statistical summaries from the data.

Through data transformation and standardization, the LFM ensures that the diverse and complex data collected from urban environments are refined into a machine-processable, consistent, and analytically valuable dataset.

##### 5.3.2.3 Data preprocessing pipeline

The data preprocessing pipeline of the LFM is designed to handle the diverse and complex characteristics of urban flow data, ensuring compatibility for training and integration into the BCA. This pipeline incorporates a series of structured steps that refine and transform raw data into standardized formats, enabling seamless integration and analysis within the model's framework. Below are detailed clarifications on the preprocessing approach, including standardization techniques, examples of data formats, and interfacing protocols for researchers.

Diverse datasets, including traffic counts, energy consumption, and waste generation, undergo systematic transformations such as normalization (e.g., min-max scaling for numerical flows) and encoding (e.g., categorical variables like waste types or vehicle categories). These preprocessing steps mitigate data variability, ensuring the model’s learning processes remain effective. Combined with spatial and temporal alignment techniques, these transformations enable seamless data integration from multiple subprojects into the LFM, supporting robust analysis and accurate predictions.

For spatiotemporal standardization, temporal data are aligned into sequences with fixed intervals (e.g., hourly or daily), while spatial data are mapped onto a unified grid format using GIS tools, ensuring consistent spatial resolution. For instance, traffic flow data from sensors are aggregated as hourly counts and mapped to a 100x100 spatial grid, while waste collection data are encoded as volume metrics per zone, aligned with the same spatial grid structure.

Standardized data formats used in the LFM include representations like traffic flow data (e.g., timestamp, grid_id, vehicle_count), energy usage data (e.g., timestamp, grid_id, energy_consumed_kWh), and waste generation data (e.g., timestamp, zone_id, waste_volume_m3, waste_type). These standardized formats ensure alignment with the LFM’s positional and temporal encoding requirements, facilitating precise analysis and prediction.

Preprocessing is an integral component of the LFM framework, featuring a modular pipeline that harmonizes raw data from diverse sources. Researchers interfacing with the LFM can utilize API endpoints or provided scripts, which specify input data requirements (e.g., format, resolution, encoding) and preprocessing configurations. For instance, traffic data can be formatted as timestamp, grid_id, vehicle_count, and pre-processed for direct ingestion into the LFM. This structured preprocessing pipeline ensures data compatibility and consistency and enhances the analytical value of the dataset, laying the foundation for robust and reliable urban flow modeling.

#### 5.3.3 The Blue City autoencoder

Training the LFM involves employing a custom Autoencoder—the BCA—a specialized DL architecture designed for City Flow modeling. As a GenAI model, the BCA leverages generative AI techniques to learn complex urban flow patterns, create meaningful representations, and simulate new scenarios. This enables the LFM to predict and analyze urban dynamics, making it a critical component of the overall framework. The current architecture of the BCA reflects the iterative prototyping process, where preliminary designs are tested and refined to handle structured and unstructured urban flow data effectively. Feedback from these tests informs ongoing improvements in its encoder-decoder structure.

The BCA is tailored to learn from these two data formats, much like autoencoders used in NLP and image recognition. However, it is specially adapted for the spatiality, complexities, and nuances of urban data flows. It consists of two main components: an encoder and a decoder. The encoder compresses the input data into a lower-dimensional representation, capturing the essential features and patterns. The decoder reconstructs the data from this compressed representation, allowing the model to learn a robust and meaningful representation of the urban data. The conceptual design being explored for the BCA is outlined below. These elements are not finalized but represent our current approach, subject to adjustment as we proceed with the implementation and experimentation phase.

• Encoder Architecture: The encoder is planned to leverage a combination of a stacked CNN for structured data (e.g., street networks or infrastructure data from OpenStreetMap) and a bidirectional transformer layer for unstructured data (e.g., temporal or semantic flows of energy, waste, people, vehicles, etc.). Positional encoding is integrated into the design to retain the spatial context of data, which is anticipated to be crucial for capturing both spatial patterns and temporal dependencies.
• Decoder Architecture: The decoder is designed to mirror the encoder, incorporating an attention mechanism to prioritize significant features derived during encoding. For example, a significant feature might include a temporal spike in traffic volume at a key urban intersection during peak hours. This feature could indicate patterns of congestion or bottlenecks that are critical for predicting future traffic flows or evaluating the impact of interventions like alternative routes or introducing low-traffic zones. Its role is to iteratively decode spatial-temporal representations, ensuring fidelity to the input distribution while remaining adaptable for extrapolated scenarios.

The envisioned architecture includes custom embedding layers to integrate metadata, such as geographic coordinates and temporal stamps. These features enhance the model's ability to generalize across diverse urban contexts. For example, a flow segment might include the latitude and longitude of a traffic sensor, allowing the model to contextualize the data spatially. Data points could include timestamps, enabling the model to capture daily or seasonal patterns, such as increased energy consumption during winter months.

We are planning to tag flow data with categories (e.g., "waste type: plastic" or "vehicle type: bus"), allowing the model to differentiate between distinct flow types and prioritize relevant features.

The latent space of the model will be tuned for high-dimensional spatial and temporal features, potentially leveraging domain-specific loss functions to align with urban flow datasets.

• The latent space is envisioned to accommodate high-dimensional spatial and temporal features, tailored for urban data. For example, urban flows like pedestrian movement may require the model to capture dependencies between proximity to amenities (e.g., parks or train stations) and time-of-day patterns. Energy consumption might require correlations between peak usage periods and building types (e.g., residential vs. commercial), emphasizing the temporal-spatial relationship in latent feature representation.
• The autoencoder's training process could employ loss functions tailored to urban data types. For example, for traffic flow data, a weighted loss function could prioritize minimizing errors in high-traffic regions (e.g., major intersections or highways) while allowing greater tolerance for low-traffic areas. The model might use a spatial continuity loss for biodiversity flows to ensure that reconstructed data preserve ecological connectivity across green corridors.

This conceptual design reflects the intended capabilities of the LFM to handle diverse data modalities and predict urban dynamics effectively. However, we recognize that specifics, including network configurations and parameter tuning, will be iteratively refined as we begin implementation and experiment with various urban data types and use cases. This iterative process will ensure the architecture adapts optimally to the requirements and challenges of urban flow modeling.

##### 5.3.3.1 Input embedding and positional encoding

**Fig. 12:**
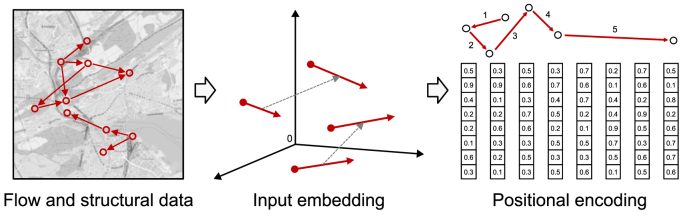
Details of the encoding process (Blue City Autoencoder).

As with ChatGPT, which predicts or generates new word sequences based on prompts, the BCA learns from abstracted city data similar to how regular autoencoders learn from text documents (word constellations) and images (pixel constellations). The BCA entails specific input embedding and positional encoding for urban flows (Fig. 12). Input embedding and positional encoding enable the LFM to extrapolate from known data and factor in the spatial and temporal dimensions that are crucial for accurate predictions. For example, training on the typical traffic flow during morning rush hours or the distribution of pedestrian movement on weekends allow the LFM to forecast changes in these patterns in response to new infrastructure developments, policy implementations, or social trends.

Input embedding transforms urban flow data into a format that DL models can efficiently process and analyze. Converting each element of urban flow data (flow fragment) into numerical representations known as vector embeddings enables the BCA can handle complex urban phenomena in a structured and analyzable manner. For instance, the distance of a particular flow fragment to a hypothetical center point of a city could be encoded as part of its vector embedding. When embedded alongside other relevant characteristics (e.g., structural data and other flow data), this spatial information provides a rich, multidimensional representation of urban flows. Such embeddings allow the LFM to discern the significance of spatial relationships and flow dependencies.

Positional encoding involves embedding positional information in space and time into the data, enabling the LFM to understand the sequence and context of urban flows. By integrating positional encoding, BCA can accurately capture the unique positional characteristics of particular city flows, such as the movement of people, vehicles, and goods, for different locations. These contextualized data allow the models to recognize patterns and dependencies in existing flow sequences. Leveraging this comprehensive understanding, the LFM can predict future urban flow sequences with increasing accuracy.

##### 5.3.3.2 Learning features

During the training process, the BCA’s primary task is to extract meaningful features, dependencies and patterns from the urban flow data. This learning process is essential for effectively representing and understanding the complex interactions and dynamics of urban flows.

• Pattern recognition: The BCA learns to identify recurring patterns and relationships within the data, such as traffic congestion trends, pedestrian movement patterns, or energy consumption cycles.
• Anomaly detection: The BCA recognizes unusual or atypical events. Identifying anomalies is important for detecting issues or opportunities for intervention in urban management.
• Dimensionality reduction: Reducing the complexity of the data to its most informative features facilitates more efficient and accurate analysis through BCA. This dimensionality reduction process is key to managing and interpreting large-scale urban data.
• Generalization Ability: the BCA develops the ability to generalize learned patterns to new, unseen data. This generalization is critical for predicting future urban dynamics based on past and present data.

##### 5.3.3.3 Generalization features

**Fig. 13:**
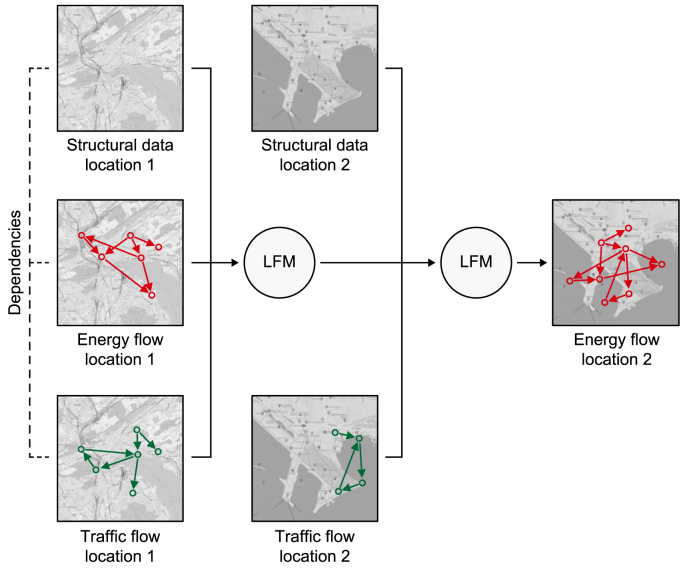
Generalization features of the large flow model: illustration of its inferring dependencies from structural, energy, and traffic flow data in a first Location 1 as a basis for estimating flow data in Location 2.

Generalization denotes the LFM’s capacity to perform effectively on new, unseen data that were not part of the training dataset, based on new contexts or stimuli (Fig. 13). This ability is fundamental for the predictive accuracy and reliability of the LFM, especially when making forecasts about future urban dynamics based on past and present data.

In the context of GSAI, the generalization ability of the LFM is of particular significance due to the dynamic and continuously evolving nature of urban environments. Cities are complex systems where conditions can change rapidly due to various factors such as economic shifts, policy changes, infrastructure developments, and societal trends.

The LFM’s ability to generalize ensures that it can adapt to new scenarios and changes within the city that were not explicitly present in the training data. For instance, the LFM can predict traffic patterns in response to stimuli, e.g., a new transportation policy or assess the impact of a new commercial development on pedestrian flows.

Generalization further enables the LFM to provide predictive insights into future urban dynamics. The model can forecast how these dynamics might evolve in response to various stimuli or changes by understanding the fundamental patterns and relationships that govern city flows. Urban data are characterized by high variability and complexity. The generalization ability of the LFM allows it to navigate this variability, ensuring that its predictions remain robust and relevant despite fluctuations in data.

Developing and maintaining a strong generalization ability in the LFM involves several key considerations:

• The training dataset should be as diverse and representative as possible, encompassing various urban conditions, scenarios, and data types. This diversity helps the model learn more universal patterns that apply to various situations.
• Implementing regularization techniques during training prevents the model from overfitting to the training data. Techniques such as variational dropout, weight decay, or early stopping ensure that the model learns general patterns rather than memorizing specific data points.
• Using cross-validation methods during model training and evaluation helps assess the model's generalization ability. Training and testing the model on different subsets of the data makes it possible to gauge how well the model performs on unseen data.
• Continuous learning and updating are essential for adapting to the dynamic nature of urban environments. Regularly updating the model with new data and retraining it will help maintain and improve its generalization ability over time.

In sum, the LFM leverages GenAI and, hence, an advanced DL architectures to generate spatially accurate representations of the urban environment, enabling planners and policymakers to simulate and analyze various scenarios for sustainable smart city development. Through its adaptive learning and predictive capabilities and data-driven insights, the LFM holds significant potential for informing evidence-based decision-making and shaping the cities of tomorrow.

### 5.4 Ongoing development progress and future endeavor and potential

The development phase of the LFM, which is currently underway, builds upon the completed design phase and focuses on the construction and refinement of the LFM. Key progress so far includes the initial integration and standardization of diverse datasets from Blue City subprojects and the iterative prototyping of the BCA. These efforts aim to establish a robust and scalable model capable of accurately representing and predicting urban flows, while also addressing the intricacies of multimodal urban data.

The datasets utilized during this phase encompass a wide range of urban flows. The integration and harmonization of these datasets are ongoing, ensuring consistency across varying formats, temporal resolutions, and spatial contexts. These processes allow the LFM to construct meaningful representations of urban systems, capturing spatial-temporal dependencies critical for planning and decision-making. Future work will build on these steps to refine the LFM. Specifically, the development phase will prioritize:

(1) Refining the BCA architecture: Enhancements will improve the encoder-decoder structure to handle high-dimensional data more effectively, incorporating advanced features such as domain-specific loss functions and expanded latent space capabilities to capture urban dynamics better.
(2) Expanding simulation modules: Simulation capabilities will be developed to model complex urban scenarios, such as traffic congestion mitigation strategies, energy optimization, or biodiversity conservation efforts, providing actionable insights for policymakers.
(3) Advancing visualization tools: Interactive tools will be created to translate the model’s predictions and simulations into user-friendly outputs, enabling stakeholders to explore urban flow scenarios visually and intuitively.
(4) Increasing scalability: The LFM will be scaled to incorporate datasets from other cities within Switzerland and beyond, allowing for broader applicability and ensuring its adaptability to diverse urban contexts and challenges.

The LFM presented in this study is designed as a foundational architecture for urban flows, trained and tested on the Blue City Project’s diverse datasets. While the model’s current scope is localized, its scalability-oriented design ensures the potential for deployment in other cities across Switzerland, Europe, and Asia. The iterative nature of its development, combined with its modular architecture, positions the LFM to evolve into a comprehensive FM. This evolution will be achieved through further training on diverse datasets, integrating new data sources (e.g., IoT sensors and networks, environmental data, socio-economic data, etc.), and implementing continuous learning techniques to keep the model adaptive and relevant.

Ultimately, these features underscore the LFM’s potential to address critical urban challenges on a larger scale. The LFM will empower urban planners, policymakers, and researchers to tackle issues such as rapid urbanization, climate resilience, and resource optimization with high precision and foresight by providing advanced tools for data-driven decision-making. As the development phase progresses toward the release of the initial prototype in 2026, the LFM continues to pave the way for more sustainable, efficient, and resilient urban environments in line with the overall goal of the Blue City Project.

### 5.5 Synthesis of the outcomes of the Blue City Project case study into a conceptual model

**Fig. 14:**
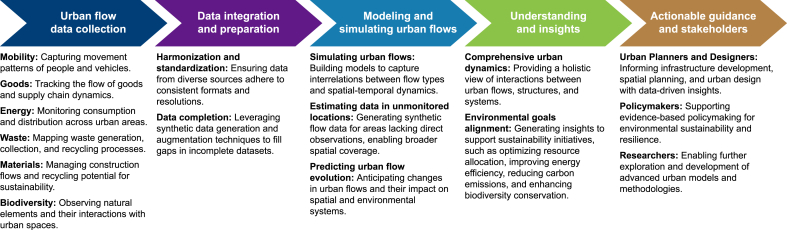
A conceptual model for the Blue City case study outcomes.

The outcomes of the Blue City Project case study have been synthesized into a conceptual model that captures the key components of urban flow modeling and simulation as applied through the LFM (Fig. 14). The model is structured around five interconnected layers, each representing critical processes and functionalities that underpin the LFM. Derived from the design and ongoing development phases of the LFM, it highlights its potential to address spatial challenges and promote environmental sustainability. This synthesis serves as a culmination of the case study findings, showcasing the relevance and applicability of the LFM to sustainable urban planning and design.

The conceptual model derived from the Blue City Project Case Study demonstrates the innovative potential of the LFM by organizing the findings into a cohesive five-layer framework. It emphasizes the importance of integrating diverse urban datasets, employing advanced modeling and simulation techniques, and generating predictive analytics to address the inherent complexities of urban environments. Harmonizing and standardizing data while focusing on actionable insights, the model offers a scalable and adaptable framework for broader applications in sustainable urban development. This synthesis underscores the LFM's practical relevance within the Blue City Project and establishes a foundation for future research and enhancements. The conceptual model represents a key step forward in developing advanced UDT tools by supporting cities in achieving resilience, and environmental sustainability.

## 6 Discussion

Urban planning and design increasingly rely on innovative technologies, such as UDT, GenAI, and FMs, to tackle the multifaceted challenges of rapid urbanization and ecological pressures. Within this landscape, this study introduces a pioneering LFM to enhance the computational and predictive capabilities of UDT frameworks. The LFM is specifically designed to support sustainable smart city planning and design, addressing critical urban dynamics and providing actionable insights in the context of the Blue City Project in Lausanne City. This section presents a comprehensive discussion of the findings, emphasizing the implications, limitations, and challenges encountered, alongside recommendations for future research and development.

### 6.1 Summary of findings and interpretation of results

The findings underscore the potential of the LFM, specifically tailored for UDT, to effectively model and analyze complex urban flows encompassing a wide array of dynamics within urban environments. The LFM enables a comprehensive understanding of spatial and temporal urban dynamics by integrating diverse data streams. Analyzing urban flows provides critical insights into how various city areas are utilized and how their usage evolves. Furthermore, the LFM can identify high-activity zones, highlighting areas of concentrated human, economic, and social activities. This is critical for the assessment of urban vitality and the optimization of resource allocation.

The findings also illustrate the LFM’s ability to inform sustainable urban planning and design by providing data-driven insights into the spatial distribution and temporal fluctuations of urban systems. Integrating these flows supports evidence-based decision-making, enabling urban planners to address environmental challenges, such as energy efficiency, emissions reduction, and ecological preservation. Moreover, the LFM's adaptability allows it to account for the dynamic nature of cities, offering a solid framework for predicting and managing future urban transformations. This study demonstrates the critical role of advanced modeling and simulation tools in enabling cities to adapt to complex and interconnected challenges. It highlight the value of augmenting UDT functionalities to create more sustainable, resilient, and efficient urban environments through adaptive learning, comprehensive data analysis, and predictive capabilities.

Building on these foundational insights, the discussion delves into the LFM's specific role in addressing urban challenges, emphasizing its adaptability and applicability within the Blue City Project. In this context, the findings emphasize the LFM’s ability to estimate flow data in unmonitored locations and predict the evolution of urban flows, showcasing its potential as a critical resource for long-term strategic planning and proactive urban management. The design phase has ensured that the LFM is scalable and flexible, enabling it to accommodate future expansions and integrate new data streams. This robustness lays the groundwork for adaptability to the ever-evolving conditions of urban environments. Moreover, the LFM offers a holistic perspective on interrelated urban systems, uncovering hidden relationships and trends that inform sustainable development strategies and interventions.

The LFM’s ability to identify correlations and causalities among various flow categories—such as traffic congestion, pedestrian and vehicle movement, energy consumption, waste generation, material usage, and biodiversity loss—provides a deeper understanding of urban dynamics. It uncovers insights into potential challenges, such as the environmental impacts of resource utilization, and identifies opportunities for sustainable development, such as optimizing energy flows. The LFM facilitates evidence-based decision-making to optimize urban planning and design processes by analyzing these interconnections.

Integrating advanced AI tools through the LFM strengthens urban resilience and sustainability by capturing spatial dynamics and flow interconnections. Planners can develop adaptive strategies to address challenges such as climate change, economic shifts, or infrastructure stress by harnessing the LFM’s detailed simulations and predictive analytics. Its application in the Blue City Project illustrates how it can inform policies and actions to create smarter, more efficient, and sustainable urban environments.

### 6.2 Comparative analysis

The introduced FM for urban flows represents a significant advancement in the field of GSAI, addressing a notable gap in existing FMs tailored for urban planning and design. Compared to previous models such as DiffusionSat [138], Prithvi [60], and GPT-4 [55], and multimodal FM for GeoAI [79], the LFM stands out for its originality, methodology, and focus on urban flows.

While models like DiffusionSat [138] focus on generative tasks for satellite imagery, the LFM specifically targets modeling urban flows in the context of UDT. The two models differ in their input data and conditioning information. The LFM incorporates metadata such as geolocation for urban flow modeling, while DiffusionSat relies on spectral information for satellite image generation. The LFM's emphasis on urban flows to understand spatial dynamics and space vitality distinguishes it from DiffusionSat's focus on temporal generation and super-resolution tasks. This distinction highlights the LFM's novelty in addressing the unique challenges of urban planning and design, where understanding spatial dynamics and space vitality are crucial factors for enhancing sustainable urban development practices.

Introduced by Jakubik et al. [60], Prithvi is a transformer-based geospatial FM trained on multispectral satellite imagery. While Prithvi and the LFM aim to address geospatial challenges, they differ in their scope and applications. Prithvi's pre-training on extensive geospatial data enables it to perform tasks such as cloud gap imputation, flood mapping, and wildfire scar segmentation. In contrast, the LFM's tailored focus on urban flows enables it to capture spatial dynamics and facilitate data-driven decision-making processes for sustainable smart city planning and design. Focusing on urban flows, the LFM fills a critical gap in GeoAI research, enabling data-driven approaches to address geospatial challenges in urban environments.

While GPT-4 [55] stands as a state-of-the-art multimodal large language model, its ability to represent geographic diversity and spatial features remains limited. In contrast, the LFM leverages urban flow data and metadata to achieve a more nuanced and comprehensive understanding of urban dynamics and their interconnections. Although both GPT-4 and the LFM incorporate spatial information, they different in their modalities and objectives. GPT-4's geo-guessing experiment highlights gaps in its capability to encode and interpret geographic features, underscoring its limitations in representing complex spatial data. Conversely, the LFM's integration of diverse urban flow datasets enables it to capture intricate urban dynamics: It provides actionable insights to support evidence-based decision-making in sustainable urban planning and design.

The introduction of the LFM fills a critical gap in GSAI and GeoAI research by providing a specialized LFM tailored for UDT and sustainable smart city planning and design. This comparative analysis highlights the diversity of approaches in GSAI and GeoAI and emphasizes the need for domain-specific FMs to address the unique challenges of geospatial applications. While both fields contribute to the advancement of GenAI in geographical and spatial domains, the LFM stands out for its focus on urban flows to advance UDT's planning and design capabilities in the context of sustainable smart cities. Its originality lies in its ability to capture spatial dynamics, space vitality, and interconnections within urban environments.

As part of the comparative analysis, the LFM stands out for its innovative urban planning and design, which addresses critical limitations inherent in traditional methodologies. It achieves this by completing impartial city data, effectively mitigating biases and gaps that have historically hindered comprehensive urban analysis. This capability ensures a more accurate representation of urban environments and sets a new benchmark for data-driven urban analytics. Furthermore, the LFM's ability to estimate flow data in new locations represents a significant advancement, enabling predictions that extend beyond the constraints of historical data. Its predictive power is further demonstrated through its capacity to forecast the evolution of flow data, equipping decision-makers with insights into future urban dynamics and supporting proactive planning and design strategies.

Furthermore, the LFM's integration of real-time data analysis enhances its ability to monitor and respond to emerging urban challenges, offering a distinct advantage over traditional approaches. Promoting a holistic understanding of urban systems through the integration of diverse datasets and advanced analytical techniques, the LFM supports evidence-based decision-making that accounts for the interconnected nature of urban environments.

### 6.3 Implication for research, practice, and policymaking

The introduction of the LFM tailored for UDT presents significant implications for research, practice, and policymaking in the field of sustainable smart city planning and design. For research, the LFM represents a groundbreaking integration of GenAI, FMs, and UDT, creating opportunities to explore the intersection of advanced technologies, urban planning and design, and geospatial analysis. Researchers can utilize the LFM to conduct in-depth studies on urban flows, spatial dynamics, space vitality, and environmental impacts. This includes examining complex relationships among flow categories, identifying emerging patterns in urban data, and testing intervention hypotheses through scenario predictions. The case study of the Blue City Project provides a valuable empirical foundation, demonstrating the practical application of GenAI and FMs in real-world urban settings. Researchers can build on these insights to refine and adapt the LFM to diverse urban contexts, addressing unique challenges in different cities and regions. Furthermore, the LFM's capability to model dynamic urban systems encourages interdisciplinary collaborations, fostering new knowledge on sustainable urban development.

For practitioners such as urban planners and designers, the LFM provides a powerful data-driven tool to analyze, simulate, and optimize urban flows. Its ability to predict the evolution of flow data and integrate multimodal datasets enhances proactive urban management, infrastructure planning, and resource optimization. Practitioners gain deeper insights into urban dynamics, space vitality, and the interactions among various urban systems by integrating the LFM into UDT frameworks. This facilitates adaptive planning strategies and evidence-based decision-making, enabling the optimization of transportation systems, energy efficiency, and material usage. The LFM also supports practical applications such as traffic management and waste optimization, helping cities achieve sustainability goals while responding to rapidly changing urban conditions.

The LFM is a critical resource for developing evidence-based policies promoting urban sustainability and resilience. Its ability to provide comprehensive analyses and scenario testing allows policymakers to anticipate the outcomes of various interventions and design resilient, sustainable urban strategies. Policymakers can ground their decisions in accurate, data-driven insights, enhancing their effectiveness in addressing challenges. Moreover, the LFM can inform strategies to tackle critical urban issues like traffic congestion, energy consumption, mobility inefficiencies, and environmental degradation. Governments can leverage the LFM to foster innovation in urban planning, improve public services, and enhance the quality of life for urban residents. 10.13039/100014337Furthermore, the model supports broader policy objectives, including climate change mitigation and economic development, reinforcing its transformative potential in shaping future urban policies.

### 6.4 The positioning, scope, and scalability of the large flow model

The design phase of the LFM demonstrates its potential as a FM architecture specifically tailored for urban flows. While the model is trained and tested on data from the Blue City Project, these datasets encompass diverse urban domains. Moreover, the collaborative involvement of city authorities, researchers, and industrial partners ensures a robust representation of urban dynamics. While localized to the Blue City Project, this diversity highlights the model’s adaptability and practical relevance to complex urban systems.

Despite these strengths, we acknowledge that the LFM in its present state does not fully meet the traditional definition of a FM, which typically involves training on vast datasets spanning multiple contexts and applications. Instead, this study focuses on the design and ongoing development of the LFM as a foundational architecture, using the Blue City Project as a proof of concept. This approach emphasizes the LFM’s ability to model urban flows comprehensively while setting realistic expectations regarding its scalability and broader applicability.

The scalability of the LFM is a core feature of its conceptualization and planning as well as construction and refinement. As part of the Blue City Project’s original plan, the model is intended for deployment in other cities across Switzerland and beyond. This planned expansion underscores the LFM’s potential to evolve into a comprehensive foundation model capable of addressing urban challenges across diverse geographical and socio-economic contexts. Future iterations will further enhance this scalability by integrating larger and more diverse datasets from various urban environments, enabling the model to meet the broader expectations of a FM by enhancing its generalizability and robustness. This will include incorporating data from cities with varying socio-economic, cultural, and environmental characteristics, ensuring the model can capture the full spectrum of urban dynamics. Moreover, the LFM’s modular architecture will enable seamless adaptation to new urban flows, technologies, and data types, further expanding its applicability.

This study lays the groundwork for the LFM’s development by presenting its architecture, demonstrating its capabilities through a case study, and setting the stage for its future evolution. The insights and applications derived from this work provide a clear pathway for the LFM to scale and contribute to sustainable urban planning and design.

### 6.5 Limitations

While the LFM demonstrates significant potential for advancing urban planning and design, several limitations must be acknowledged and addressed. One of the LFM's primary objectives is to address the challenge of incomplete or impartial data by generating synthetic content and employing data augmentation techniques. Through its GenAI capabilities, the LFM learns patterns and relationships from existing datasets to simulate missing data points, producing realistic and contextually accurate synthetic data. However, a notable limitation of this generative approach is its dependency on the quality and comprehensiveness of the input data. The accuracy and contextual relevance of the synthetic data heavily rely on the diversity, quantity, and reliability of the foundational datasets used for training. When input data are inconsistent or insufficient, the LFM risks amplifying these deficiencies, potentially leading to skewed analyses or flawed predictions. Moreover, critical challenges remain to validate the synthetic data against real-world conditions and ensure their alignment with dynamic and unique urban contexts. Addressing these concerns is essential for maintaining the reliability and applicability of the LFM in diverse urban environments.

The computational complexity of the LFM poses a challenge, particularly concerning scalability in large urban areas or high-resolution datasets. Processing and analyzing vast quantities of spatiotemporal data demands significant computational resources, which may not be accessible to all stakeholders. Moreover, the model's performance varies across urban contexts, reflecting differences in infrastructure, demographics, and environmental conditions. Although the LFM demonstrates strong performance in specific districts or cities, its ability to generalize across diverse urban settings may necessitate further customization and adaptation to ensure broader applicability. The reliance on a single case study—the Blue City Project in Lausanne City—introduces potential contextual bias, as its unique characteristics may not represent the complexities of other urban environments. Expanding the evaluation to multiple case studies would enhance the LFM's generalizability and robustness. Finally, while accuracy and precision are commonly used to assess the LFM, they may inadequately capture its effectiveness in addressing complex urban challenges or driving sustainable urban planning and design. More comprehensive metrics are needed to evaluate the model's broader impact on sustainable urban development practices.

Lastly, the preprocessing and conditioning of input data, including integrating metadata such as geolocation and temporal information, may inadvertently introduce biases or errors that affect the model's outputs. Ensuring the reliability, consistency, and accuracy of these inputs is crucial for maintaining the validity of the LFM's predictions and analytical outcomes. Addressing these limitations will be critical for refining the LFM's robustness and expanding its applicability to diverse urban contexts.

### 6.6 Challenges

Concerning challenges, the development and deployment of FMs for urban flows, despite their numerous benefits, raise critical concerns and pose potential risks in different domains, including urban planning and design. These issues, as also relevant to GenAI models (e.g., Ref. [9,[139], [140], [141], [142], [143], [144], [145]]), include the following, as identified in various studies (e.g., Ref. [32,37,61,[78], [79], [80], [81]]):

*Bias and fairness*: Both FMs and GenAI models are trained on vast datasets that may embed historical biases, leading to unfair outcomes and perpetuating inequalities, particularly for marginalized communities. In urban planning and design, biased models could reinforce existing disparities in access to resources, services, and opportunities. For example, if historical data contains biases against specific demographic groups, the model's decisions and predictions could disproportionately disadvantage these groups. Ethical considerations include identifying and mitigating biases in training data, ensuring fairness in model outputs, and implementing frameworks for equity-driven design.

*Transparency and accountability*: The complexity and black-box nature of FMs and GenAI models make it challenging to understand their decision-making processes. This lack of transparency can undermine trust and accountability, especially in decisions that affect urban communities and infrastructure. Ethical considerations emphasize the need for explainable AI techniques and transparent workflows that allow stakeholders to understand, evaluate, and challenge model outcomes.

*Privacy and surveillance*: GenAI and FMs, trained on large-scale urban datasets, can inadvertently capture sensitive information about individuals and communities. In urban planning and design, there is a risk of privacy infringement and unintended surveillance if these models analyze personal or location-based data without consent. Ethical measures should include robust data anonymization techniques, informed consent protocols, and privacy-by-design principles to safeguard individual and community data.

*Security and vulnerabilities*: GenAI and FMs are susceptible to adversarial attacks, where malicious inputs can manipulate model outputs. In urban planning and design, such vulnerabilities could jeopardize critical infrastructure or public safety by producing misleading or harmful recommendations. Ensuring robust security measures, such as adversarial training and continuous vulnerability assessments, is essential to protect these systems and ensure their resilience against cyber threats.

*Environmental costs*: The significant computational resources required to train and deploy GenAI models and FMs contribute to substantial carbon emissions and energy consumption, raising environmental concerns. This is especially pertinent given the increasing demand for these technologies and their implications for climate change. Ethical considerations include exploring energy-efficient architectures, leveraging green computing practices, and aligning AI development with sustainability goals.

*Societal impacts*: The widespread adoption of GenAI and FMs can lead to profound societal changes, such as job displacement, cultural shifts, and altered power dynamics. In urban planning and design, ethical considerations involve assessing the broader implications of AI integration, such as its effects on social equity, public participation, and governance. Conducting comprehensive societal impact assessments ensures AI adoption prioritizes societal well-being and mitigates potential negative consequences.

Addressing these challenges requires a multifaceted approach involving interdisciplinary collaboration, active stakeholder engagement, and strict adherence to ethical guidelines and frameworks. City stakeholders can harness the transformative opportunities of FMs and GenAI while proactively mitigating risks and minimizing potential harms by fostering cooperation among urban planners, engineers, computer scientists, data scientists, policymakers, and community representatives. Ensuring these technologies contribute to a more sustainable and equitable urban future necessitates embedding ethical principles into every stage of their development and deployment. This includes prioritizing transparency, fairness, accountability, and inclusivity to align technological advancements with broader societal and environmental goals.

### 6.7 Suggestions for future research directions

While the current LFM at this stage relies on data from a specific large-scale project, its architecture has been intentionally designed to allow further training on broader datasets. Future research should aim to expand its training scope, explore its integration with UDT frameworks, and advance its capabilities to align more closely with the characteristics of a FM.

Several areas warrant attention to address identified limitations and advance the field. First, improving the quality, reliability, and availability of urban flow data is crucial. This effort may involve developing advanced data collection methods, leveraging emerging technologies, and fostering collaborations with stakeholders to integrate diverse and representative datasets. Such initiatives are essential for addressing the LFM's dependency on high-quality input data, ensuring it can still perform effectively in scenarios where data quality or availability is limited, and reducing the risk of amplifying biases in synthetic data generation, thereby enhancing the model's reliability and applicability. Second, research should explore innovative approaches to model development and validation to address the computational challenges and improve the interpretability of the LFM. This includes designing validation mechanisms to ensure that synthetic data aligns with real-world conditions and dynamic urban contexts, mitigating the risk of skewed analyses or flawed predictions. Third, scaling up the application of the LFM to larger and more diverse urban areas is key. This includes integrating it into existing urban planning and design frameworks through collaboration with city governments, urban planners, and other stakeholders to co-create solutions that tackle real-world urban challenges and contribute to building more sustainable, inclusive, and resilient cities.

Given that the LFM is still under development, ongoing research efforts should prioritize iterative testing, validation, and refinement. This process can be enhanced through experiments and real-world applications, particularly in Lausanne City, which serves as a test bed and living lab for the Blue City Project. Current UDT frameworks remain inadequate in handling the complexity of urban systems [67,68,146] and often lack proper validation mechanisms [66,147]. Establishing continuous feedback loops and fostering collaboration with researchers and practitioners will facilitate iterative improvements and accelerate the adoption of the LFM in urban planning contexts, ensuring its relevance and impact.

To address data bias and representation challenges, future research should focus on curating diverse and inclusive training datasets that represent various demographic groups, geographic regions, and socio-economic contexts. Data augmentation techniques and fairness-aware training methodologies can further mitigate biases and enhance model performance. Improving the transparency and explainability of the LFM is equally important. Researchers should develop tools for auditing model predictions, identifying biases, and ensuring alignment with ethical guidelines during model development and deployment.

Future research should focus on developing more efficient architectures and training techniques to address scalability challenges and resource demands. Techniques such as knowledge distillation, model pruning, and quantization can optimize the model's size and computational requirements, enabling it to handle larger datasets and broader urban contexts without sacrificing performance. In addition, implementing energy-efficient training methodologies and resource-monitoring strategies can reduce the environmental impact of training and deploying the LFM. These advancements will ensure that the LFM remains both scalable and accessible to a diverse range of stakeholders while supporting its application in varied and resource-constrained urban environments.

Expanding evaluation beyond a single case study to address contextual bias is also critical. Future research should explore applying the LFM to multiple urban settings, allowing for more robust evaluation and enhancing its generalizability across diverse contexts. This will ensure that the unique characteristics of Lausanne City do not overly influence findings and that the model’s applicability to a broader range of urban environments is validated.

Finally, interdisciplinary collaborations between AI researchers, urban planners, ethicists, policymakers, and community stakeholders are imperative to address challenges and risks associated with FMs. Such partnerships can ensure that societal implications are considered, ethical concerns are addressed, and responsible AI systems are developed. Researchers and practitioners can, by pursuing these strategies, harness the transformative potential of GenAI and FMs while mitigating associated risks, ultimately advancing the responsible deployment of GenAI and FM technologies in urban planning and design.

## 7 Conclusion

This study introduced a pioneering LFM, developed with GenAI capabilities, grounded in a robust foundational framework, and designed for integration into UDT systems to enhance their computational and predictive functionalities. The LFM addresses critical spatial challenges and supports sustainable smart city planning and design by advancing the capabilities of UDT. Using the Blue City Project in Lausanne City as a case study, this research illustrated the model's potential to tackle the complexities of urban flows and multimodal dynamics. It demonstrated its practical relevance and significance in the context of UDT.

The findings underscore the LFM’s ability to effectively capture and analyze urban flows, including mobility, goods, energy, waste, materials, biodiversity, and information. These flows are critical for understanding spatial dynamics, assessing space vitality, and enabling data-driven decision-making processes that advance sustainable urban development. The empirical insights from the Blue City Project validate the tangible benefits of the LFM, showcasing how it addresses challenges in city modeling, simulation, and strategic planning. Integrating adaptive learning, generative, and predictive capabilities, the LFM expands the functionality of UDT frameworks, offering a transformative approach to modeling spatial dynamics and urban interconnections. This equips urban planners, designers, policymakers, and researchers with valuable insights and tools to foster innovation and create sustainable urban strategies and designs.

The LFM distinguishes itself through its innovative approach to tackling critical urban planning and design challenges. It achieves this by completing impartial and incomplete city data, estimating flow data in previously unmonitored locations, predicting the evolution of urban flows, and offering a comprehensive understanding of urban dynamics and their interconnections. The LFM enables more accurate analyses and generates actionable insights by addressing persistent spatial challenges. Furthermore, it paves the way for new opportunities to overcome obstacles in the broader development and implementation of UDT frameworks, ultimately laying the foundation for effective, adaptive, and sustainable urban management practices that drive progress in urban planning and design.

This study makes several significant contributions to urban planning and design and the development and implementation of UDT frameworks through the design and operationalization of the LFM. The key contributions are as follows:

*Development of a robust FM for urban flows:* The study introduces a pioneering LFM specifically designed for urban environments. This model is a critical tool for urban planners and decision-makers, providing a reliable framework for managing and analyzing complex urban systems.

*Enhanced data integration and interoperability:* The study develops methodologies for harmonizing diverse datasets from various subprojects within the Blue City Project, addressing challenges related to data quality, standardization, and interoperability. The LFM ensures seamless data utilization within UDT frameworks, enabling comprehensive analysis and informed decision-making, by integrating data from various domains.

***Completing city data with synthetic data and augmentation****:* The LFM addresses critical gaps and biases in urban datasets through the use of synthetic data generation and data augmentation techniques. These capabilities enhance the comprehensiveness and accuracy of urban system representations, mitigating the limitations of traditional data collection methods. The LFM provides a more holistic and unbiased representation of urban flows by generating realistic synthetic data and augmenting incomplete datasets. This, in turn, enables more accurate modeling, analysis, and decision-making processes.

*Improved predictive capabilities for urban planning*: The LFM enhances the predictive capabilities of UDT by accurately estimating flow data in unmonitored locations and forecasting the evolution of urban flows. This predictive power equips urban planners to anticipate changes, allocate resources strategically, and proactively address potential urban challenges, improving the resilience and sustainability of urban environments.

*Holistic understanding of urban dynamics:* The LFM provides a comprehensive view of urban dynamics and interconnections, enabling planners to identify synergies between urban systems and optimize resource allocation. This holistic approach supports integrated urban planning and management, fostering more coordinated and effective strategies.

*Enhanced sustainable urban development:* The LFM facilitates strategies that minimize environmental impact and promote efficient resource use. Integrating data from critical urban systems helps planners design interventions that align with environmental sustainability goals, creating more livable and eco-friendly urban environments.

The integration of the LFM into UDT frameworks represents a significant advancement in data-driven urban planning and design, equipping stakeholders with the tools and insights needed to navigate the complexities of modern urban environments. However, it introduces several challenges that must be carefully addressed to ensure its successful and sustainable implementation. Addressing these challenges requires interdisciplinary collaboration among urban planners, engineers, computer scientists, data scientists, policymakers, and community stakeholders. This collective effort is crucial for co-creating decision-making systems that are equitable, transparent, and accountable, while ensuring the responsible use of the LFM in urban planning and design. Establishing ethical guidelines and governance mechanisms is essential to prevent potential misuse or unintended consequences. While the LFM holds immense potential for transforming sustainable smart city planning and design through UDT, its success ultimately depends on tackling these challenges and ensurinalig its adaptability to the dynamic and ever-evolving nature of urban environments.

## CRediT authorship contribution statement

**Jeffrey Huang:** Writing - Review & Editing, Writing - Original Draft, Visualization, Validation, Software, Project Administration, Investigation, Formal Analysis, Data Curation, Conceptualization. **Simon Elias Bibri:** Writing - Review & Editing, Writing - Original Draft, Visualization, Software, Methodology, Investigation, Formal Analysis, Data Curation, Conceptualization. **Paul Keel:** Writing - Review & Editing, Writing - Original Draft, Visualization, Software, Investigation, Formal Analysis, Data Curation, Conceptualization.

## Declaration of competing interest

The authors declare that they have no known competing financial interests or personal relationships that could have appeared to influence the work reported in this paper.
